# Programming photoactuation by modulating the degree of charge-separation in DASA-based liquid crystal elastomers

**DOI:** 10.1039/d6sc05729b

**Published:** 2026-10-06

**Authors:** Arnab Nandi, Jesus Guillen Campos, Waseem Shindy, Javier Read de Alaniz

**Affiliations:** a Department of Chemistry and Biochemistry, University of California Santa Barbara CA 93106 USA javier@chem.ucsb.edu

## Abstract

Photoresponsive soft actuators enable light-driven shape changes with spatial and temporal control, typically achieved by integrating molecular photoswitches into liquid crystal elastomers (LCEs). Commonly, the structure–property relationship of photoswitches is well explored in dilute solution phase. However, connecting solution-phase molecular electronic structure to function in crosslinked LCEs remains challenging. Here, we use donor–acceptor Stenhouse adducts (DASAs) as a tunable photoswitch family to engineer an electronically diverse DASA-LCE platform with varied charge separation. A series of five DASAs spanning neutral to strongly zwitterionic character were incorporated into siloxane-based LCEs *via* late-stage functionalization. The solution-phase solvatochromic slope serves as a spectroscopic descriptor for ground-state push–pull character and reveals a strong linear correlation with solid-state reversible photofading rate coefficients, establishing a unique molecular-level predictor for DASA-based LCE switching. Increasing charge separation produces a continuum of LCE responses: neutral DASAs undergo efficient photoisomerization that drives reversible uniaxial contraction. In contrast, zwitterionic DASAs drive photothermal bending, enabling bilayer actuators with biomimetic crawling (0.2 cm s^−1^) and jumping. These results establish a design rule linking DASA electronic structure to macroscopic actuation and provide a practical screening metric for white-light-responsive LCE actuators.

## Abstract 2

Photoresponsive liquid crystal elastomers (LCEs) enable light-driven shape changes with spatial and temporal control.

## Graphical abstract

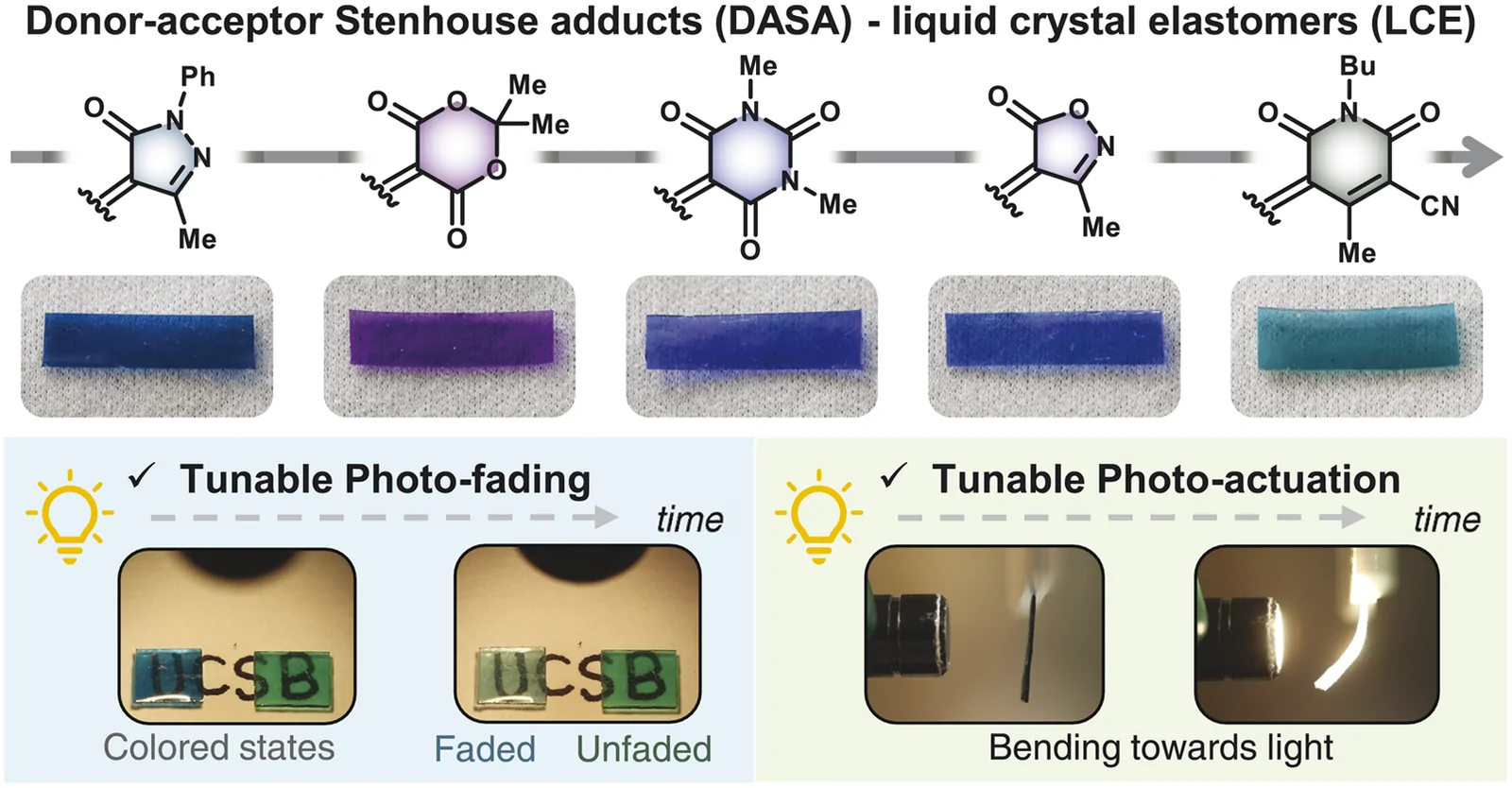

## Introduction

Liquid crystal elastomers (LCEs) combine the macroscopic elasticity of polymer networks with the long-range orientational order characteristic of liquid crystals.^1–5^ As a result, LCEs undergo reversible transitions that enable anisotropic, programmable deformations in response to external stimuli.^6–8^ Programmability is perhaps the defining feature that distinguishes LCEs from other soft materials. Through control of mesogen alignment, complex deformation modes – bending, twisting, elongation – can be encoded directly into the material. Emerging fabrication techniques, including direct ink writing and photopatterning, allow multimodal responses in increasingly complex geometries.^4,9–14^ Among the available stimuli used to actuate LCEs, light is particularly attractive because it enables non-invasive, reversible actuation with high spatial and temporal precision.^15–22^ White light, although less commonly employed, offers broad spectral overlap with many light absorbing derivatives. In most cases, these light-driven responses are achieved by incorporating molecular photochromes that convert photon absorption into changes in molecular geometry, heat, or polarity, ultimately driving macroscopic deformation.^23^

Selection of the photochromic motifs can add another layer of programmability to the LCE actuation by coupling their intrinsic switching to the macroscopic light-driven response. For example, Aprahamian and Katsonis demonstrated that bistable hydrozone-based photoswitches can be incorporated into liquid crystal polymer networks to drive shape transformations. Here, the light-induced geometries of the material, in the macroscopic scale, exhibited long-term stability that mirrors the inherent biostability of the molecular switch.^24^ In a complementary strategy, Zeng and Priimagi reported an elegant system that combined azobenzene for UV-induced shape programming coupled to a photothermal dye to enable shape morphing under 660 nm irradiation.^18^ The synergistic integration of photochemical and photothermal processes allowed a single actuator to access six distinct, reconfigurable states. Despite these and other notable examples,^25,26^ programming control over actuation behavior with molecular level control remains challenging, often necessitating full optimization of LCE fabrication conditions to explore different photoresponsive molecules.

Among the available classes of photochromic molecules, donor–acceptor Stenhouse adducts (DASAs) represent an attractive platform for programmable light-driven LCE-based soft actuators.^22^ DASAs are negatively photochromic molecules that undergo reversible “fading” transitioning from a strongly colored open form to a colorless closed form upon irradiation, followed by thermal recovery in the dark (Fig. 1). This negative photochromism enables unprecedented light penetration depths of up to 5.5 mm—length scales that are inaccessible to conventional UV-activated photochromes.^20^ Importantly, structural variations of the carbon-acid acceptor preserve this negative photochromic response while modulating key molecular parameters, including switching kinetics and the degree of charge separation or zwitterionic character within the push–pull framework.^22^ While a number of structure–property relationships have been established for small-molecule DASA adducts in solution,^27,28^ the influence of charge separation, and the resulting switching kinetics within LCEs remains unexplored.

**Fig. 1:**
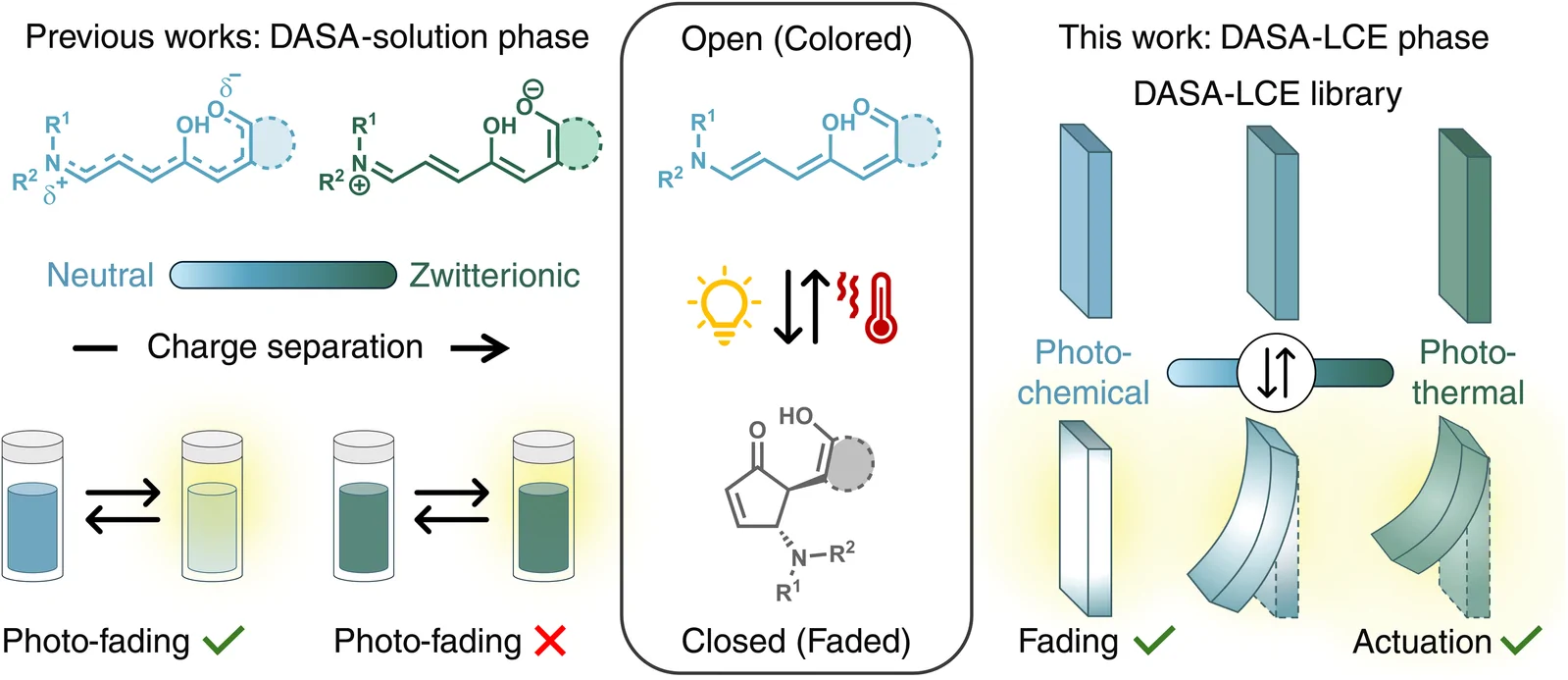
Transition from characterized DASA photochemistry in solution to charge-separation-driven behavior in LCEs. The schematic highlights how integrating DASAs into LCEs introduces new pathways for tunable, light-driven actuation *via* photo-chemical and photo-thermal pathways.

To address this challenge, we recently developed a late-stage functionalization strategy that enables the modular incorporation of DASAs and related photoresponsive motifs into LCEs using Finkelmann's two-stage hydrosilylation method.^20,29–31^ This approach provides donor-functionalized LCEs in which DASA-based photochromes can be constructed by reacting with the appropriate acceptor motif without altering the network architecture. In this work, we establish a DASA-LCE platform that directly correlates solvatochromic slopes obtained from solution-phase absorption spectra with solid-state photoswitching and macroscopic actuation. This work bridges the gap between molecular photochemistry and material performance, providing both a fundamental design framework for DASA-based photochromic systems and a practical pathway toward the rational development of advanced, programmable light-powered LCEs.

## Results and discussion

### Design and synthesis of small molecule DASAs

To investigate the relationship between molecular architecture, push–pull charge separation behavior, and photochemical function, we sought to leverage the donor 4-methoxy-*N*-(pent-4-en-1-yl)aniline originally used by Bruns and Boesel and our group.^20,32^ The prior works established this donor was compatible with hydrosilylation chemistry. Coupling the donor with a broad-range of activated furan-acceptors within the aligned LCE is adopted to realize a series of DASAs with progressively increasing zwitterionic character. To achieve this, we selected methyl-pyrazolone (M-Pyra), meldrum's acid (Mel), barbituric acid (Barb), methyl-oxazolone (M-Oxa), and hydropyridine (HDP) as the acceptor moieties. The donor and the furan-acceptors were synthesized according to reported literature procedures.^33–35^ The range of acceptors enabled systematic tuning of the degree of ground-state push–pull across the series, ranging from neutral to strongly zwitterionic character (Fig. 2). Full synthetic procedures and characterization are described in the SI.

**Fig. 2:**
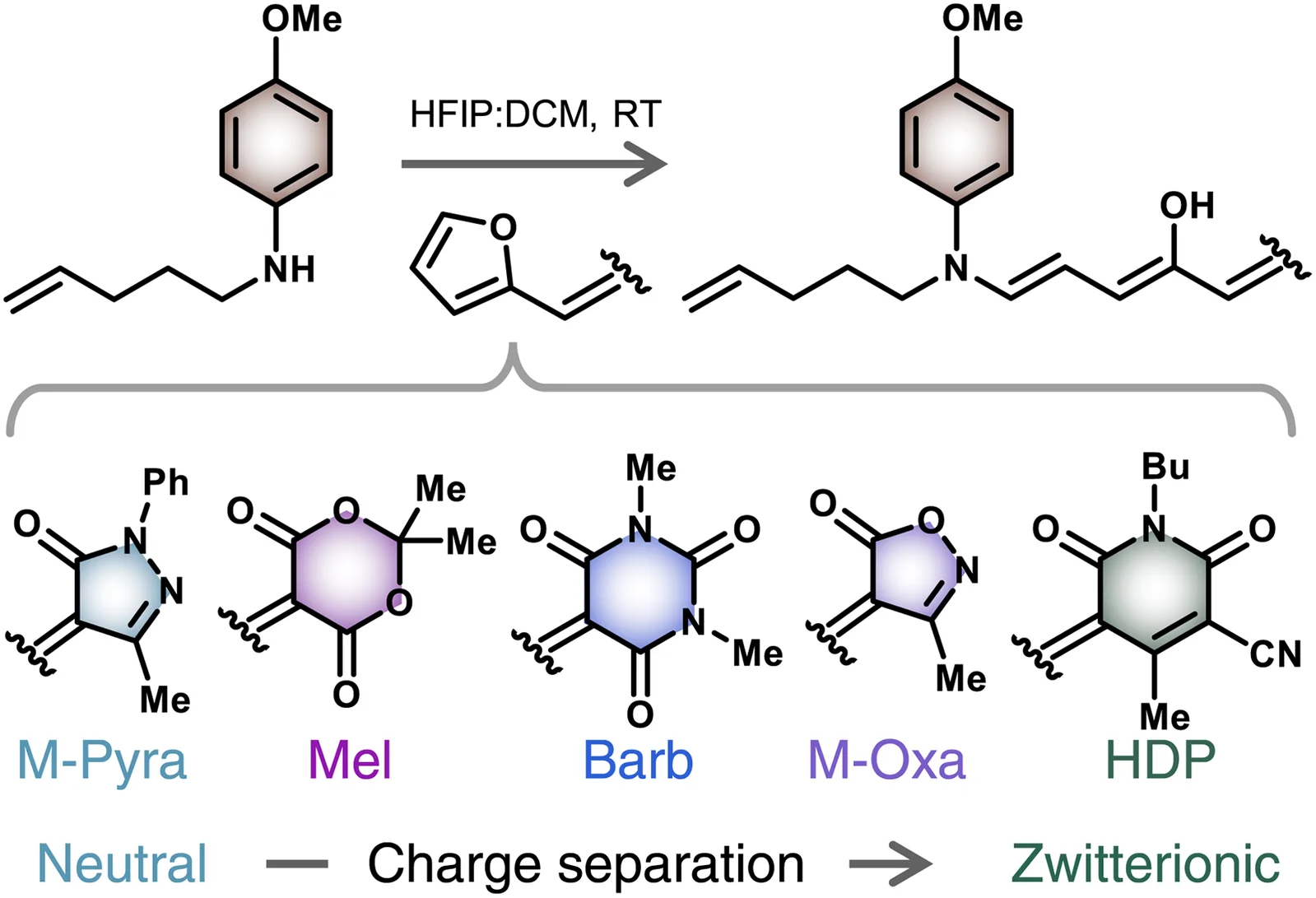
General synthesis scheme of the representative small molecule DASAs.

### Solvatochromic analysis of small molecule DASAs

To determine the electronic character of the DASAs synthesized, the ground state charge separation was evaluated through solvatochromic analysis. Here, shifts in absorption maximum across solvents of varying polarity provide a quantitative proxy for ground-state push–pull magnitude.^27^ Absorption spectra of the five small molecule DASAs were collected initially in toluene, ethyl acetate, chloroform, and acetonitrile, with additional solvents included in the SI (Fig. S1–S8). The magnitude of solvatochromic behavior across the series is most clearly captured by the contrast between M-Pyra and HDP; M-Pyra displays a negligible blue shift of only 2 nm from 592 nm (toluene to acetonitrile), while HDP undergoes a pronounced blue shift of 50 nm from 652 nm (toluene to acetonitrile) with increasing solvent polarity (Fig. 3A). Solvatochromic slopes were obtained by linear regression of *λ*_max_ against solvent polarity parameters (Fig. 3B); a steeper negative slope indicates greater stabilization of the charge-separated ground state by polar solvents, reflecting more pronounced zwitterionic character, whereas a slope approaching zero is consistent with a predominantly neutral ground-state electronic structure.^27^ The full slope plots and absorption traces for the remaining derivatives are provided in the SI. As summarized in Fig. 3C, the series spans a wide range of solvatochromic responses, from −3 nm for M-Pyra to −94 nm for HDP, with intermediate values of −12 nm (Mel), −22 nm (Barb), and −29 nm (M-Oxa). In previous literature, low solvatochromic slopes have been correlated with better photoswitching in a range of solvents and polymer matrices. Conversely, DASAs exhibiting high solvatochromic slopes show minimal or no photoswitching under the same conditions.^27,32,36^

**Fig. 3:**
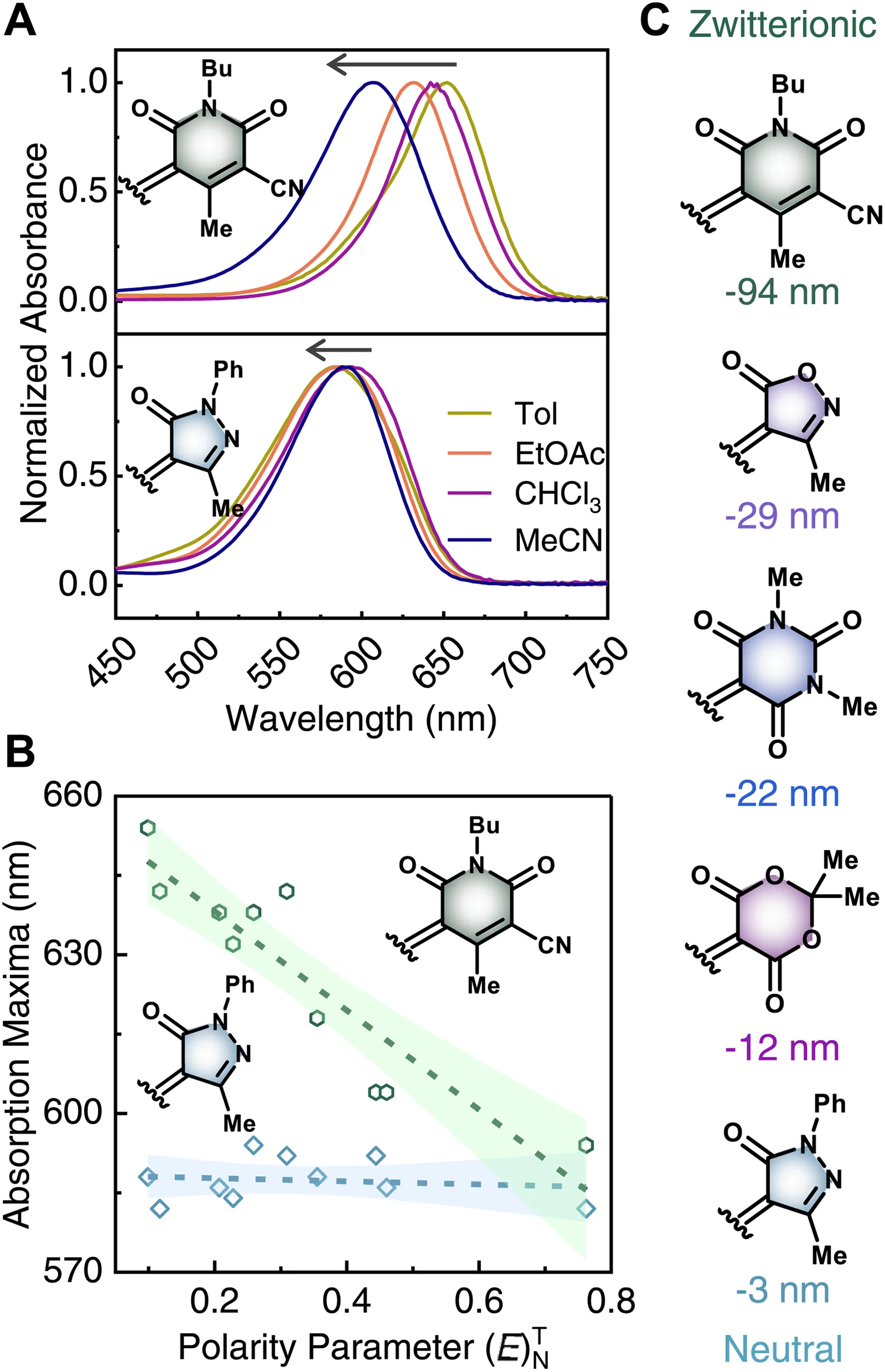
(A) Absorption spectra of HDP (top) and M-Pyra (bottom) in 4 varied polarity solvents. (B) Solvatochromic slope determination from *λ*_max_ against solvent polarity parameter plot. (C) List of the solvatochromic slope values for all five representative DASAs showing charge separation increasing from bottom to top.

### DASA incorporation into liquid crystal elastomers

To ensure comparable DASA loading across the LCEs studied here, the kinetics of DASA formation within the LCE network were characterized for each acceptor by time-dependent absorption measurements, establishing the timescales over which incorporation reached completion. However, to avoid complications that might arise from *in situ* crosslinking during these kinetic studies, a model liquid crystal polymer was prepared by hydrosilylation of polymethylhydrosiloxane (PHMS), the mesogenic unit, and the donor in the absence of a crosslinker, to yield a linear LC polymer bearing pendant donor amine groups (Fig. 4A). The stoichiometric ratio of the LCE formulation was maintained consistent throughout the different kinetic analysis. Aliquots of the LC polymer were combined with solutions (25 mg mL^−1^ concentration) of each acceptor in DCM : HFIP (9 : 1), and the evolution of the characteristic DASA absorption peak was monitored over 60 min (Fig. 4B). HDP, bearing the strongest electron-withdrawing acceptor, exhibited the fastest and highest conversion, approaching 80% conversion after 60 minutes. The moderately electron-withdrawing derivatives followed with progressively slower kinetics: M-Oxa, M-Pyra, Barb reached normalized absorbance values of only 0.6, 0.3 and 0.28 respectively, at 60 minutes, indicating incomplete conversion at this timescale. Mel showed virtually no conversion within 60 minutes (Fig. 4B). This trend is consistent with the increased electrophilicity of the furan-acceptor across this series, which facilitates nucleophilic addition of the donor amine. Note, the 60 minutes reaction time was selected to highlight the difference in reactivity among the acceptors rather to achieve maximum conversion with the model system. For the LCE films investigated in this study, films were reacted for 16 h to ensure maximum conversion. This reaction time was selected based on UV-vis studies that showed conversion reached a plateau following overnight reaction (Fig. S9). The synthesis of the donor-functionalized linear model polymer and the UV-vis kinetics protocol were described in the SI.

**Fig. 4:**
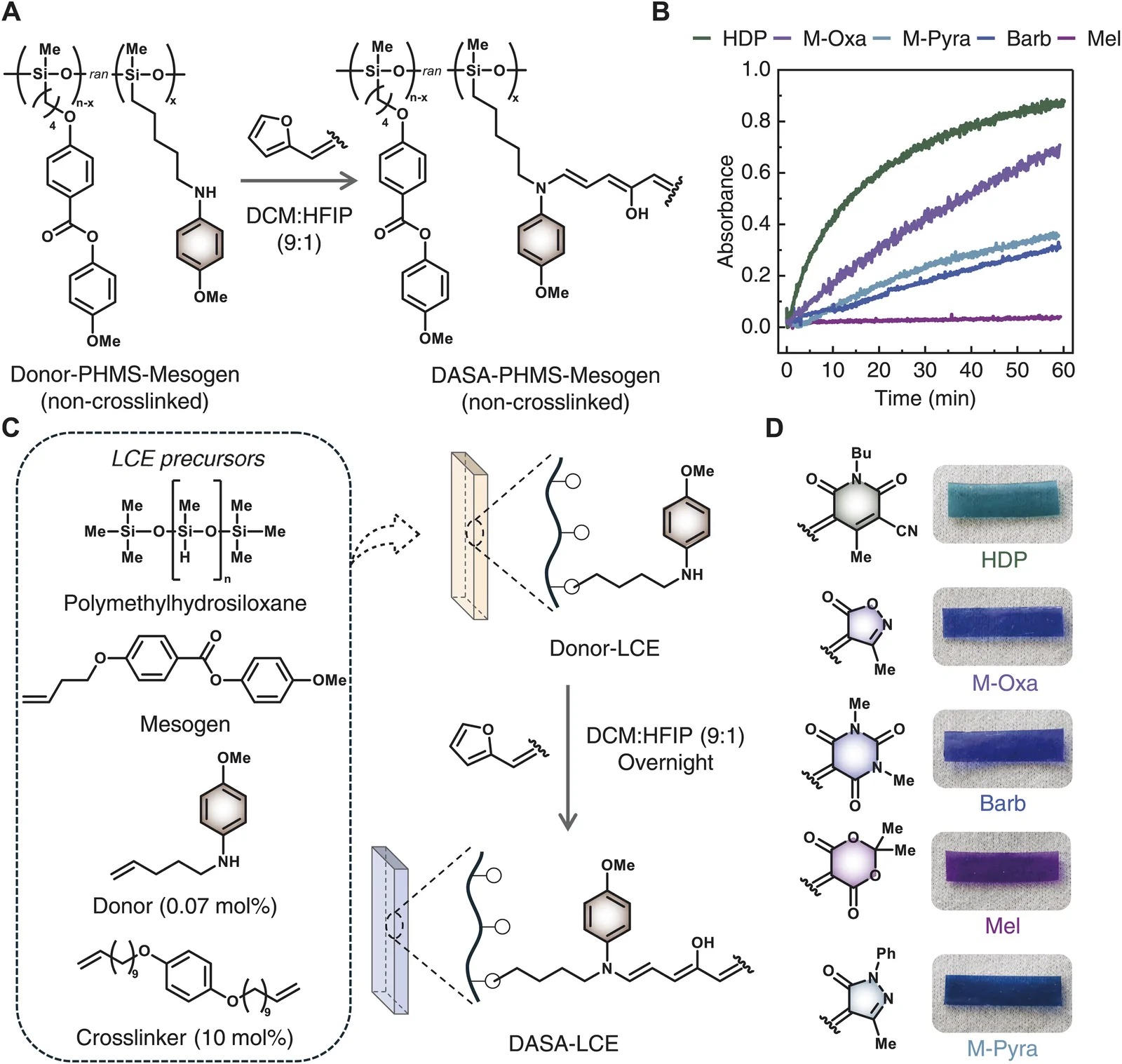
(A) Model siloxane-donor LC linear polymer for DASA-fication in concentrated acceptor solutions for kinetics study. (B) Time dependent DASA evolution in the model siloxane-donor LC assembly for 60 min using absorption spectroscopy. (C) Schematic representation of the late-stage functionalization strategy for preparing DASA-LCEs: donor-functionalized LCEs undergo post-polymerization reaction with various carbon acid acceptors *via* swelling in furan adduct solutions. (D) Images of all the DASA-LCE films displaying distinct colors.

Guided by these kinetic benchmarks, each DASA was incorporated into side-chain siloxane-based LCEs consisting of 10% crosslinking *via* Finkelmann's two-stage method (Fig. 4C).^20,30^ The fabrication method of the LCEs is described in the SI (Scheme S1). In the first state, the lightly crosslinked networks were formed by hydrosilylation of PHMS with the mesogenic unit, the donor (0.07 mol%) and a crosslinker (10 mol%). The molar feed of the mesogen, donor, and crosslinker was formulated to stoichiometrically match the total Si–H functionality on PHMS, to enable complete consumption of the reactive Si–H sites during network formation. Under mechanical alignment, the material continues to crosslink which locks the nematic monodomain orientation. The mechanical properties of the resulting donor-LCE network were characterized by uniaxial tensile testing prior to DASA functionalization; the elastic modulus, determined from the slope of the linear region of the stress–strain curve (0–10% strain, *R*^2^ = 0.99), was found to be 1.56 ± 0.07 MPa (Fig. S10).

DASA incorporation was achieved by swelling the donor-functionalized LCE films overnight in concentrated solutions of the corresponding furan–carbon acid adducts (25 mg mL^−1^ in 9 : 1 DCM : HFIP), reaching each derivative's maximum attainable DASA conversion within the theoretical ceiling of 0.07 mol% loading (based on 0.07 mol% donor functionalization). Given its sluggish DASA formation kinetics, Mel-LCE required an extended swelling period of three days to achieve conversion comparable to the other derivatives by UV-vis. Following vacuum drying, all five DASA-LCE films (thickness of ∼0.4–0.5 mm) displayed intense, uniform coloration across thickness (Fig. S11), visually confirming chromophore incorporation across the series (Fig. 4D). While the present protocol does not permit precise, quantitative control over the final DASA concentration achieved in each network, establishing such uniform control across derivatives is an important target for future work. For the present study, reaching a substantial DASA concentration sufficient to reliably trigger light-driven actuation in each LCE was sufficient to establish the structure–property relationships explored here.

The molecular order of the DASA-LCE network was characterized by wide-angle X-ray scattering (WAXS) on aligned films (Fig. S12). The 1D and 2D diffraction pattern shows a diffuse wide-angle reflection at *q* = 1.39 Å^−1^ (*d* = 4.52 Å), concentrated in equatorial arcs perpendicular to the director, arising from lateral packing of the mesogens, along with a weak, broad feature at *q* ≈ 0.30 Å^−1^ (*d* ≈ 21 Å) corresponding to the extended length of the side group (≈20–21 Å from the siloxane backbone to the methoxy terminus). Both features are diffuse rather than sharp, consistent with the short-range longitudinal correlation characteristic of a nematic phase. The orientational order parameter obtained from the azimuthal intensity distribution of the wide-angle reflection was found to be *S* = 0.69–0.70 across the series, in agreement with the nematic order previously reported for this polysiloxane-based system.^20,37^ The thermal properties of each DASA-LCE were characterized by differential scanning calorimetry (DSC). Films exhibited nearly identical thermomechanical behavior, with glass transition temperatures (*T*_g_) of 6–8 °C and nematic-isotropic phase transition temperatures (*T*_ni_) of 75 and 78 °C (Fig. S13), consistent with the narrow range of transition temperatures previously reported for DASA-LCEs at comparably low DASA loading.^20^ These results support that DASA incorporation at 0.07 mol% does not significantly perturb the bulk elastomer network. This indicates that differences in photomechanical response across the series can be attributed primarily to the electronic character of the incorporated DASA, and thus photoswitching properties of DASA, rather than variations in network architecture.

### Photoswitching properties of DASA-LCEs

The spectroscopic behavior of the DASA-LCE series is best understood in the context of the DASA photoswitching mechanism, where negative photochromism and competing thermal relaxation pathways together govern the observed absorption response. Under visible light irradiation, the colored open isomer (A) undergoes a rapid actinic step to yield the primary photoproduct (B), followed by sequential bond rotation (B′) and electrocyclic ring closure to afford the colorless closed isomer (C) (Fig. 5A). Efficient net conversion to C requires the forward reaction to outcompete the thermal reversion from C to B/B′ and A. This equilibrium establishes the photothermal stationary state (PTSS). At high light intensities, photothermal heating arising from DASA absorption shifts this equilibrium toward the colored open form, creating a self-reinforcing feedback loop that suppresses ring closure and formation of C.^22,27,38^

**Fig. 5:**
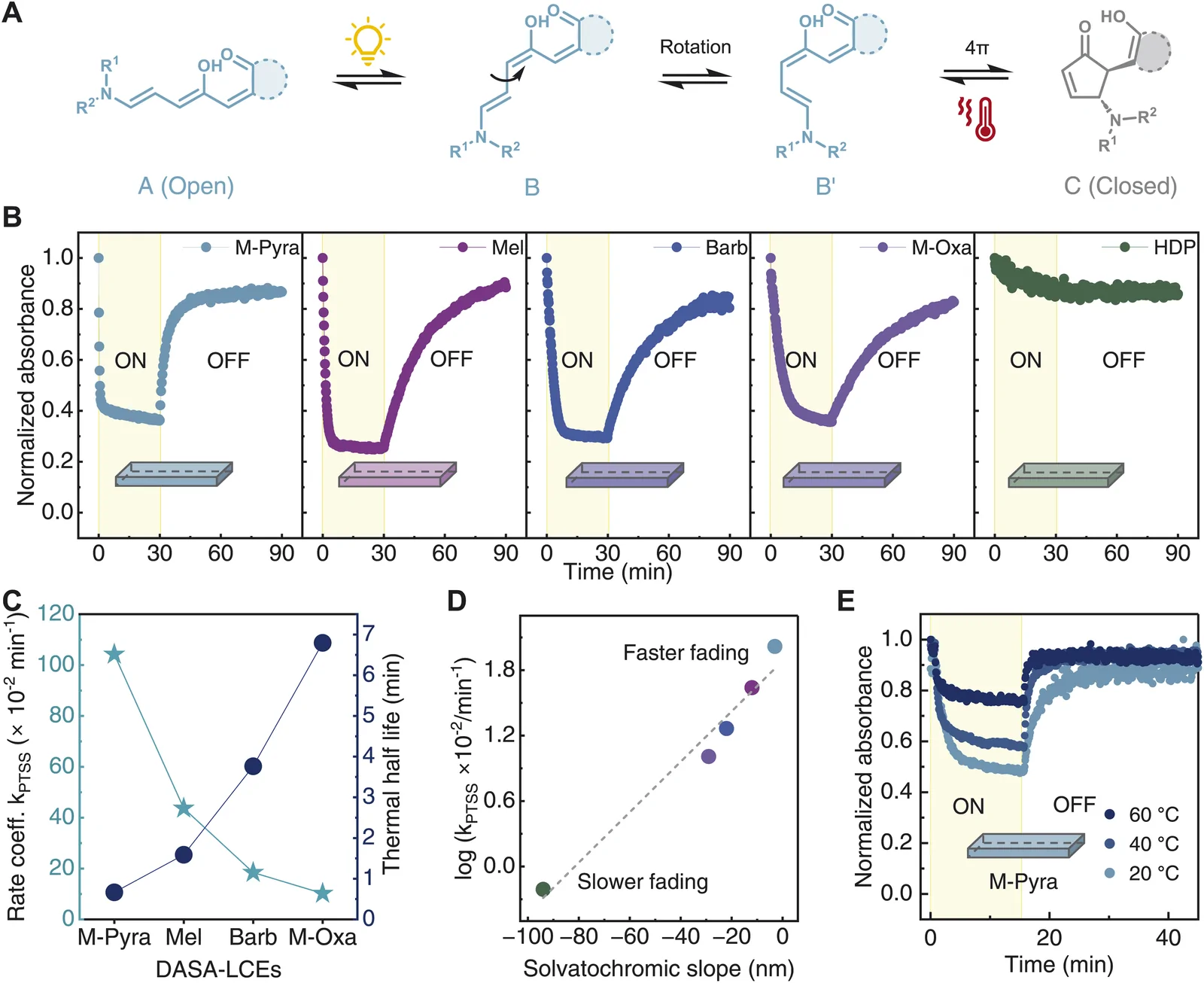
(A) Simplified diagram illustrating DASA photoisomerization mechanism, highlighting light-responsive and nonlight-responsive intermediates. (B) Photo-fading and recovery kinetics of DASA-LCEs under light; pronounced fading for neutral and fading resistance for zwitterionic DASAs. (C) Fading rate constants and thermal half-lives of DASA-LCEs. (D) Linear correlation between rate coefficient in LCE phase and solvatochromic slope from small molecule studies. (E) Temperature dependent photo-fading experiment for M-Pyra-LCE at three different temperatures.

Based on this framework, we sought to correlate the dynamic photo-fading behavior of DASA-LCEs under light irradiation with the ground-state push–pull electronic character of molecular DASA photochrome. Absorption spectra of all five DASA-LCEs were recorded at room temperature. The series displayed *λ*_max_ absorption profiles spanning from 569 nm (Mel-LCE) to 645 nm (HDP-LCE) with the remaining DASA-LCEs in between (Fig. S14 and Table S1). This variation arises from the push–pull electronic character of the triene chromophore, which simultaneously governs both the absorption maximum and the solvatochromic slope. A broadband visible light was used for all measurements, as all five DASA-LCEs exhibit some degree of spectral overlaps in this region, enabling consistent activation conditions across the series (emission profile shown in Fig. S15). Future work will focus on decoupling *λ*_max_ from the solvatochromic slope, which would allow narrow excitation wavelength to be employed without sacrificing photochemical efficiency.

To characterize photoswitching kinetics, the LCE films were studied using a transient pump-probe absorption spectrometer under dark conditions to prevent stray-light activation. Films were irradiated with low-intensity white light (120 mW cm^−2^; 0.5 mW cm^−2^ at 550 nm and 0.3 mW cm^−2^ at 650 nm) for 30 min to monitor photofading, followed by a 60 min dark phase to assess thermal recovery. Upon irradiation, M-Pyra, Mel, Barb and M-Oxa-LCEs each showed absorbance decay at their respective absorption maxima, consistent with forward isomerization to the closed, colorless form (Fig. 5B); the time-resolved traces followed first-order exponential decay profiles (Fig. S16). All of the DASA-LCEs (except HDP) reached PTSS within 30 min with M-Pyra being the fastest and M-Oxa being the slowest among them. This trend is reflected in the rate coefficients: M-Pyra exhibited the fastest photofading (*k*_PTSS_ = (104.2 ± 12.5) × 10^−2^ min^−1^), nearly an order of magnitude higher than Mel, Barb and M-Oxa. The slower approach to PTSS for Barb relative to our previous report using monochromatic irradiation is consistent with the reduced spectral overlap between broadband white light and the Barb DASA absorption profile.^20^ In contrast, HDP-LCE was photofading-resistant (only ∼10% absorbance loss), and thus did not have a measurable thermal recovery; it is therefore omitted from Fig. 5C. During the dark phase, thermal recovery followed the reverse trend. M-Oxa exhibited the longest thermal half-life (*t*_1/2_ = 6.8 min), approximately eight times that of M-Pyra (0.7 min) (Fig. 5C). Full *k*_PTSS_ and *t*_1/2_ values for all five DASA-LCEs are provided in Table S2.

The kinetic trends across the series can be rationalized by the competition between the forward photoreaction and thermal back-relaxation. For the more neutral M-Pyra, the forward reaction is fast and dominates, yielding rapid and efficient photofading. As push–pull character increases across the series, the thermal back-reaction increases, progressively reducing the rate and extent of photofading until, in the strongly zwitterionic HDP-LCE, it suppresses photofading almost entirely.

The inverse correlation between push–pull electronic character and rate coefficient *k*_PTSS_ was further quantified by plotting rate coefficients against the solvatochromic slope values determined from small molecule studies (Fig. 5D). A strong linear relationship (*R*^2^ = 0.96) was observed, demonstrating that solution-phase solvatochromic slopes provide quantitative prediction of photofading kinetics that translate directly from solution to the LCE matrix. Because rate coefficients in this context are direct proxies for the activation free energy of the photochemical process, we used the Eyring equation to calculate the effective free energy barrier for electrocyclic ring closure (Δ*G*^‡^). The equations used for calculations with the determined free energy values are described in the SI (Table S2). Δ*G*^‡^ scaled linearly with solvatochromic slope across the series (Fig. S17): progressing from M-Pyra (Δ*G*^‡^ = 19.9 kcal mol^−1^) to HDP (22.9 kcal mol^−1^) raises the effective ring-closure barrier by 3 kcal mol^−1^, driven by the increasing ground-state push–pull character of the open form. To our knowledge, this is the first demonstration that a solution-phase spectroscopic parameter quantitatively predicts photofading behavior in the LCE phase, providing a rational design rule for selecting DASA acceptors tailored to specific actuation requirements.

Next, we probed how thermal relaxation kinetics impacted photoswitching performance within the LCE matrix. We examined the temperature dependence of the PTSS and Fig. 5E highlights a representative example for M-Pyra-LCE. Under continuous irradiation, the normalized absorption minimum was strongly temperature-dependent: at 20 °C the normalized absorbance reached 0.50, reflecting efficient photofading, whereas at 40 °C and 60 °C the PTSS rose to 0.60 and 0.80, respectively. This behavior is consistent with thermally accelerated back-reaction outcompeting the forward isomerization as temperature rises, shifting the open/closed equilibrium back toward the open form. Critically, thermal relaxation rate does not merely govern the kinetics of color recovery—it determines the photothermal properties within the DASA-LCE. As the photothermal stationary state increases, more fading occurs, which limits the total amount of photon energy that can be converted to heat and enables a tunable temperature gradient. These self-regulatory properties arise from control of the PTSS. Collectively, these results establish that the ground-state push–pull electronic character of the DASA, quantified by the solvatochromic slope, enables programming both the rate of photofading and the temperature sensitivity of the PTSS, providing a predictive framework for designing DASA-LCEs with tailored photothermal actuation profiles.

Triggering the photoswitching of DASA within the LCE film was expected to modulate both the optical and mechanical properties. Initially, we focused on the photofading behavior by comparing 0.5 cm × 0.7 cm × 480 µm films of M-Pyra-LCE and HDP-LCE under identical irradiation conditions (Fig. 6). Prior to irradiation, both films display intense uniform coloration characteristic of their respective open-isomer ground states—M-Pyra-LCE appearing deep blue and HDP-LCE appearing teal (Fig. 6, *t* = 0 s). Upon white light (emission profile Fig. S18) irradiation (300 mW cm^−2^; 5 mW cm^−2^ at 550 nm and 7 mW cm^−2^ at 650 nm; 3 cm from film) for 240 s, the two films respond in strikingly divergent ways: M-Pyra-LCE undergoes pronounced photofading, becoming nearly transparent, while HDP-LCE remains fully colored and unchanged from its pre-irradiation appearance (Fig. 6, *t* = 240 s). By cycling between irradiation being ON and OFF, the letter “U” in UCSB can reversible be revealed and hidden.

**Fig. 6:**
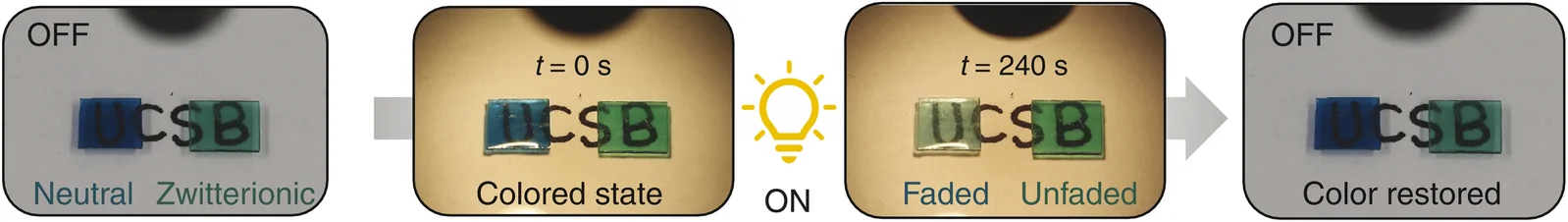
M-Pyra-LCE (neutral) and HDP-LCE (zwitterionic) are shown in their colored states before irradiation (*t* = 0 s), after 240 s of white light exposure, and following light removal.

### Photomechanical characterization of DASA-LCEs

Having successfully measured the photoswitching properties in LCEs, we next explored the photomechanical response of the DASA-LCEs (Fig. 7A). As expected, when the LCE is heated above the *T*_ni_ transition temperature it exhibits reversible uniaxial contraction, confirmed by clamping the film vertically and irradiating from the side, under which M-Pyra-LCE contracted along its length while remaining straight (Fig. 7A, top left), as well as indicated by the dotted reference box in the front view demonstration (Fig. 7A, bottom left). Of note, no measurable out-of-plane deformation was observed. This behavior is notably distinct from azobenzene- and spiropyran-based photochromic LCEs,^39–41^ in which photochemical isomerization drives bending through asymmetric order parameter changes across the film thickness. We hypothesize this mechanism is largely absent in M-Pyra-LCE due to the negative photochromic properties of DASA enabling a more homogeneous interaction of light throughout the thickness of the material. In contrast, HDP-LCE dissipated absorbed photon energy as heat and, over the same 30 seconds of irradiation period, underwent both contraction and pronounced out-of-plane bending (3D actuation). This out-of-plane bending was directly confirmed in the side-view demonstration, in which the vertically clamped HDP-LCE film curled visibly out of the plane of the film toward the light source (Fig. 7A, top right), consistent with expansion perpendicular to the nematic director and curling along the film edge observed in the front-view demonstration (Fig. 7A, top right). Bending in HDP-LCE is attributed to a through-thickness temperature gradient generated at the irradiated surface, which locally exceeds the nematic-to-isotropic transition temperature (75–77 °C) and drives asymmetric actuation. Notably, the degree of out-of-plane bending decreases progressively with increasing neutral character across the series, consistent with the trend of enhanced photofading in weaker push–pull DASAs and the negative photochromic properties within the LCE.

**Fig. 7:**
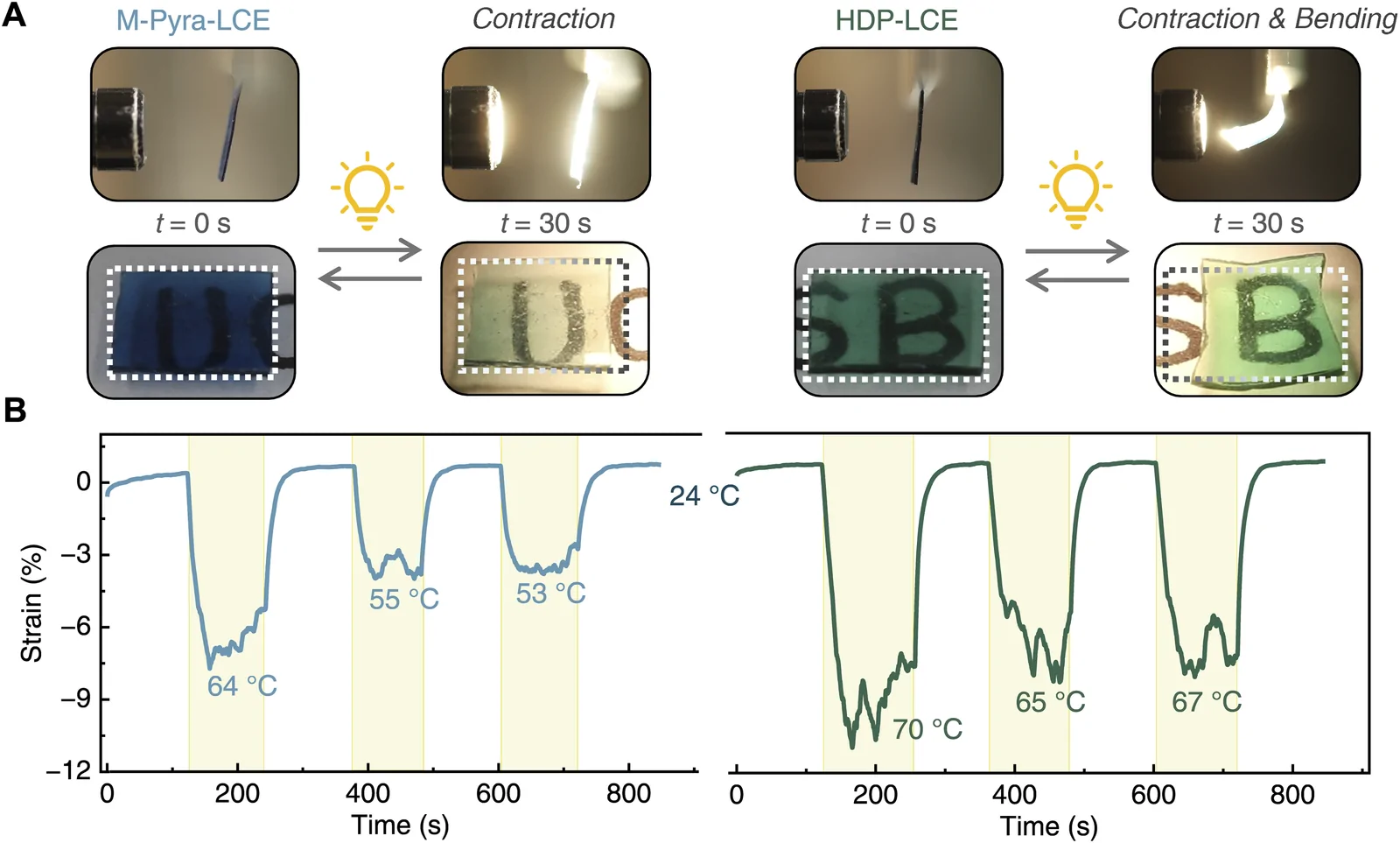
(A) Top row: demonstration in air; bottom row: demonstration on a surface. M-Pyra-LCE showing reversible contraction and HDP-LCE showing contraction coupled with bending upon irradiation. (B) Strain traces under cyclic irradiation (yellow bars) with IR camera temperature readouts at maximum strain for M-Pyra-LCE and HDP-LCE.

The photothermal contribution to actuation was quantified by monitoring surface temperature over three consecutive light-ON/light-OFF cycles (2 minutes each) using an IR camera, while photomechanical strain was simultaneously measured by dynamic mechanical analysis (DMA) under a constant applied force of 5 mN (Fig. 7B). The reproducibility of both strain profiles and surface temperatures across all three cycles confirms the robustness of each actuation mode. M-Pyra-LCE generated a maximum photomechanical strain of 7%, with surface temperatures reaching 64 °C during the first cycle and stabilizing at 53–55 °C in subsequent cycles; the modest reduction in maximum strain to 5% in later cycles is attributed to incomplete thermal recovery during the light-off phase. HDP-LCE, by contrast, generated a higher maximum strain of 12%, reflecting the sustained chromophore absorption that drives continuous photothermal contraction, with surface temperatures reaching 70 °C in the first cycle and remaining elevated at 65–67 °C across subsequent cycles. In both cases, the surface temperature rise occurred within 3–5 s of irradiation onset. The higher strain output of HDP-LCE underscores the efficiency of continuous light absorption as a driver of mechanical deformation in strongly zwitterionic DASA-LCEs; furthermore, its photofading resistance results in minimal attenuation of actuation strain across repeated cycles. The coupled DMA-IR thermometry analysis was extended to the remaining DASA-LCEs in the series, with corresponding strain and temperature traces provided in the SI (Fig. S19–S21), along with representative photothermal images (Fig. S22). The other derivatives exhibited strain values and surface temperatures comparable to, but consistently lower than, those of M-Pyra-LCE. This trend is attributed to the superior spectral overlap of M-Pyra DASA with the white light emission profile, enabling more efficient photon harvesting and greater network contraction per unit irradiance.

### Harnessing photothermal effects of HDP-LCEs for crawling and jumping actuators

Soft actuator applications targeting autonomous locomotion and environmental navigation require motion primitives such as bending, crawling, and jumping. To demonstrate the practical utility of a photothermally active DASA-LCE, we prepared a bilayer actuator that was constructed by laminating HDP-LCE with optically transparent tape as a passive layer (Fig. 8A). This asymmetric architecture exploits the mechanical mismatch between the negative thermal expansion of the HDP-LCE along its nematic axis and the negligible thermal expansion of the tape, generating a significant strain gradient across the bilayer thickness that converts contraction along nematic direction into macroscopic bending upon irradiation.

**Fig. 8:**
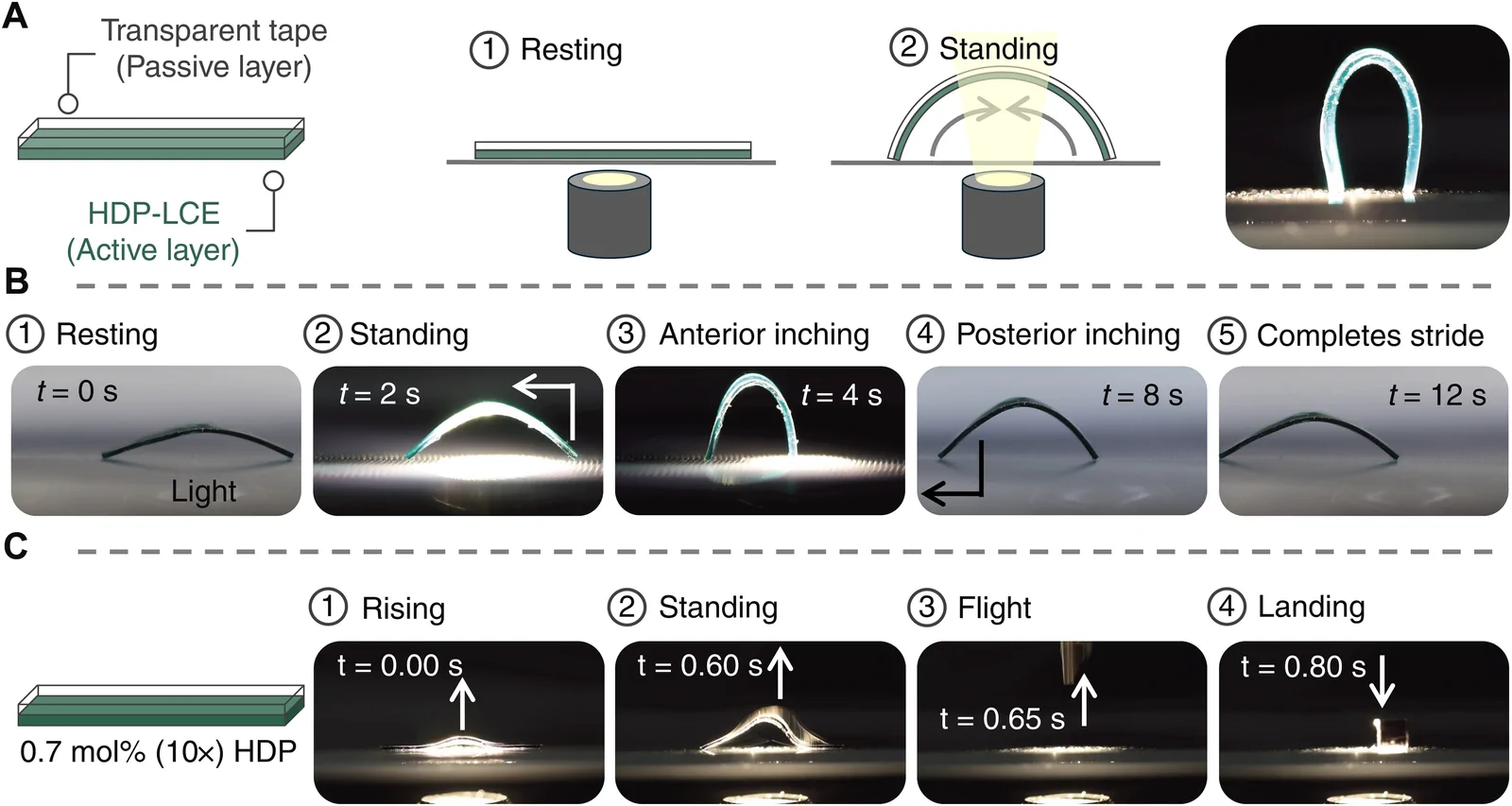
(A) and (B) Scheme and time-lapse images showing light-driven standing and crawling of HDP-LCE/transparent tape bilayers (Movie S1). (C) Light-powered jumping of a bilayer with 0.7 mol% (10×) DASA concentration (Movie S2).

Under illumination from below with the LCE facing the light source, contraction of the HDP-LCE layer drives the entire bilayer to arch upward into a near-complete loop before relaxing back to its original flat geometry upon removal of the light source (Fig. 8A). When this behavior is executed on a grooved substrate, the surface geometry imparts directional asymmetry to the actuation cycle: under illumination, the anterior end lifts and advances, while upon switching the light off, the posterior end follows. These sequential cycles combine into a biomimetic crawling gait with a recorded locomotion velocity of approximately 0.2 cm s^−1^ (Fig. 8B, *t* = 0–12 s; Movie S1). The lateral displacement of the actuator during each bending and recovery cycle, indicated by the directional arrows, confirms net translational motion across the substrate.

Pushing the system further, the DASA loading in the HDP-LCE was increased tenfold (from 0.07% to 0.7%) to amplify the photothermal response and access a qualitatively distinct actuation mode. IR thermal imaging confirmed that this elevated loading substantially increases the photothermal response, with the bilayer reaching a surface temperature of 106 °C within 5 s of irradiation compared to 24 °C at rest (Fig. S22). We hypothesize that at elevated DASA concentrations, the high surface temperatures generated during irradiation softened the LCE sufficiently to induce temporary adhesion at the bilayer edges. This soft adhesion resists standing at the center of the actuator while both ends remain pinned to the substrate, allowing elastic strain energy to accumulate under continued illumination. When the stored elastic energy exceeds the adhesion force, the bilayer is released abruptly, launching the actuator off the surface entirely in a rapid, spring-like jump (Fig. 8C; Movie S2). The sequential phases—standing (*t* = 0.00 s), elastic energy storage and adhesion (*t* = 0.60–0.65 s), snap-through release and flight (*t* = 0.65 s), and landing (*t* = 0.80 s)—mirror a light-powered high-jump completed on a sub-second timescale, a regime not typically accessible to standard photoresponsive polymer actuators.

These results demonstrate that acceptor design can be used to control energy dissipation pathway of DASA-based LCEs, giving rise to two discrete actuation profiles: neutral DASAs favor photofading-driven, reversible uniaxial contraction with full color recovery, while zwitterionic DASAs promote photothermal heating that enables coupled contraction-bending and higher strain outputs such as jumping. This distinction enables application-specific materials selection, where neutral DASA-LCEs are ideally suited for precise, optically readable linear actuation (*e.g.*, microfluidic valves), and zwitterionic DASA-LCEs are better matched to large-amplitude, continuous actuation behaviors such as light-driven gripping and autonomous locomotion.

## Conclusions

In summary, this work establishes ground-state charge separation as a DASA design handle that simultaneously governs photoswitching kinetics and photomechanical function in DASA-incorporated liquid crystal elastomers. A series of five DASAs spanning neutral to strongly zwitterionic character were incorporated into siloxane-based LCEs *via* a late-stage functionalization strategy. Solvatochromic analysis of the small-molecule series revealed a clear, monotonic gradient of push–pull character, and DASA-fication kinetics in model LC polymer assemblies confirmed that acceptor electron-withdrawing strength directly controls the rate of photoswitch formation in the polymer matrix. Photoswitching kinetics of the DASA-LCEs showed that the rate coefficient for photothermal stationary state formation decreases monotonically with increasing zwitterionic character, a trend quantitatively captured by a linear correlation between log(*k*_PTSS_) and solvatochromic slope. This establishes solution-phase solvatochromic analysis as a predictive screening metric for LCE photoswitching behavior. Coupled DMA-IR thermometry revealed that these kinetic differences translate directly into divergent actuation modes: neutral DASA-LCEs favor photoisomerization-driven reversible uniaxial contraction, while zwitterionic DASA-LCEs generate photothermal heating that enables contraction with pronounced out-of-plane bending and higher strain output. The photothermal actuation mode was further leveraged in bilayer HDP-LCE actuators capable of directional crawling and high-jumping on sub-second timescales. Collectively, these results provide a molecular-to-macroscopic framework linking DASA electronic structure to programmable light-driven mechanical behavior, with broad implications for the rational design of light-driven soft actuators.

## Author contributions

Arnab Nandi: conceptualization, investigation, synthesis and characterization, data curation, writing – original draft. Jesus Guillen Campos: conceptualization, synthesis and characterization, formal analysis, writing – review and editing. Waseem Shindy: synthesis and characterization and data curation. Javier Read de Alaniz: conceptualization, funding acquisition, superversion, writing – review and editing.

## Conflicts of interest

There are no conflicts to declare.

## Supplementary Material

SC-OLF-D6SC05729B-s001

SC-OLF-D6SC05729B-s002

SC-OLF-D6SC05729B-s003

## Data Availability

All experimental procedures and data related to this study can be found in the supporting information (SI). Supplementary information: experimental details, synthesis, NMR spectra, IR, HRMS, and additional characterization data. See DOI: https://doi.org/10.1039/d6sc05729b.
