# Supplementary material for: Programming photoactuation by modulating the degree of charge-separation in DASA-based liquid crystal elastomers
Source: Chem Sci. 2026 Oct 6. Online ahead of print. doi: 10.1039/d6sc05729b (PMC13666557; doi:10.1039/d6sc05729b)
Supplement: Supplementary file 1 [file SC-OLF-D6SC05729B-s003.pdf]

Supplementary Information (SI) for Chemical Science.
This journal is © The Royal Society of Chemistry

Programming Photoactuation by Modulating the Degree of Charge-Separation in DASA-Based Liquid Crystal Elastomers

Arnab Nandi,† Jesus Guillen Campos, Waseem Shindy and Javier Read de Alaniz

A. Nandi, J. Guillen Campos, J. Read de Alaniz.

Department of Chemistry and Biochemistry, University of California, Santa Barbara. Santa Barbara, California 93106, United States.

E-mail: jalaniz@chem.ucsb.edu

Table of Contents

1. General Experimental....3
1.1 Chemicals....3
1.2 Instruments and methods 3
1.3 Synthesis of DASA small molecules. 6
1.4 Kinetics of DASA-Fication in Linear Model Polymer....8
1.5 Synthesis of DASA LCE films by Finkelmann's method....9
1.6 Fabrication and operation of HDP-LCE bilayer actuators....11
2. Results....12
References: 28

1. General Experimental

1.1 Chemicals.

All commercially-obtained reagents were purchased from Ambeed, TCI America, Sigma Millipore or Fisher Scientific and were used without purification. Anhydrous solvents were obtained from Fischer Scientific or from a solvent purification system. Room temperature reactions were carried out between 22–25 °C. Unless stated otherwise, reactions were conducted under an air atmosphere. Analytical thin-layer chromatography (TLC) was carried out with Merck silica gel 60 F254 glass TLC plates and visualized using a combination of UV light (254/ 366 nm) and potassium permanganate or *p*-anisaldehyde. Flash column chromatography was performed with silica gel from Sigma Aldrich (technical grade, 60 Å pore size, 40–63 μm particle size). All chromatographic solvents were of ACS grade and used without further purification.

1.2 Instruments and methods

^{1}H NMR spectra were collected on a Bruker Avance III HD 400 MHz using $CDCl_3$. Data for ^{1}H NMR spectra are reported as follows: chemical shift (d ppm), multiplicity, coupling constant (J, Hz) and integration. Abbreviations for the peak multiplicities are s (singlet), d (doublet), dd (doublet of doublet), t (triplet), q (quadruplet) and m (multiplet). ^{13}C NMR spectra were recorded on Bruker Advanced NEO 500 MHz (125 MHz). Data for ^{13}C NMR spectra are reported in terms of chemical shift (d ppm).

FTIR was measured with a Thermo Nicolet iS10 FTIR Spectrometer equipped with a Smart Diamond attenuated total reflectance (ATR) accessory.

Two-dimensional wide-angle X-ray scattering (WAXS) measurements were collected with the BioPACIFIC Materials Innovation Platform SAXS/WAXS diffractometer, using an Excillum MetalJet D2+ source coupled to a Dectris EIGER2 4M hybrid photon-counting detector (2068 × 2162 pixels, 75

× 75 μm). Ga Kα radiation (λ = 1.34 Å) was used with a sample-to-detector distance of 140 mm and a beam size of 800 × 800 μm. DASA-LCE films were mounted and measured at room temperature and pressure with an exposure time of 120 s and a scattering vector range of q = 0.14–3.5 Å^{-1}. A background frame was collected under identical conditions and subtracted from each 2D pattern prior to integration.

Detector module gaps, the beamstop, and the direct-beam region were masked, then azimuthal integration over the wide-angle halo at q ≈ 1.4 Å^{-1} was performed to give the azimuthal intensity profile I(χ), using the following methodology as described by Sims et al.[1] Baseline correction was performed using the baseline-offset method, averaging the intensity within 50 pixels of the integration limits and ±5° of the director. The orientational order parameter S was calculated via the Lovell-Mitchell method (equation 1), with the Kratky expansion (equations 2–3) used for cross-verification:

$$S = -2\,\frac{\square_{0}^{\square/2} I(\square)\,\frac{3\cos^2\square - 1}{2}\,\sin\square\; d\square}{\square_{0}^{\square/2} I(\square)\,\sin\square\; d\square} \qquad (1)$$

$$I(\square) = f_0 + \frac{1}{2} f_2 \cos^2\square + \frac{3}{8} f_4 \cos^4\square + \frac{5}{16} f_6 \cos^6\square + \frac{35}{128} f_8 \cos^8\square + \dots \qquad (2)$$

$$\square\cos^2\square\square = \frac{\square_{n=0}^{\square} \frac{f_{2n}}{2n+3}}{\square_{n=0}^{\square} \frac{f_{2n}}{2n+1}}, \qquad S = \frac{3\square\cos^2\square\square - 1}{2} \qquad (3)$$

where f_{2n} in equations 2–3 are the coefficients of the orientational distribution function f(β), with β being the angle between the molecular long axis and the director. It should be noted that the Lovell–Mitchell method as reported in literature defines χ = 0° as parallel to the director, while the Kratky method defines χ = 0° as orthogonal to the director.

The aligned LCE gave S = 0.69 - 0.70, indicating good macroscopic alignment.

Optical microscopy to take images of LCE cross-sections was performed on a Keyence VHX-5000 microscope.

Optical images and videos were captured using a Canon Rebel T7 DSLR camera with a Canon EF 100 mm f/2.8 USM Macro lens.

Thermal/photothermal images were captured using a FLIR E54 handheld thermal camera.

Differential Scanning Calorimetry was measured from –20 °C to 100 °C at a temperature ramp of 3 °C/min on a TA Instruments Q2000 DSC with 50 position autosampler. Sensitivity < 0.2 μW, and baseline drift < 10 μW.

Dynamic mechanical analysis was performed on a TA Instruments DMA 850. Temperature controlled experiments were done at a constant ramp of 3 °C/ min while holding the sample at 0.005N of constant force. Light dependent strain measurements were performed under a constant strain of 0.005N and the samples were irradiated with diffuse white light.

Solution UV-Vis absorbance spectra were recorded on an Agilent 8453 UV-Visible Spectrophotometer G1103A and a ThermoScientific Genesys 50 spectrophotometer.

The photoinduced optical absorption kinetics were measured on a pump-probe setup. The pump beam was generated by a light emitting diode (LED) source (Thorlabs) coupled into a multimode optical fiber terminated with an output collimator. The LED intensity was controlled through a digital-to-analog converter (National Instruments USB-6009) using LabVIEW. The probe beam was produced by High Power MINI Deuterium Tungsten Halogen Source w/shutter 200–2000 nm (Ocean Optics DH-MINI) coupled into a multimode fiber with an output collimator for the light delivery. The probe light was modulated by a shutter (Uniblitz CS25) which could be controlled manually or through a digital output port (National Instruments USB-6009) using LabVIEW. Pump and probe beams were overlapped using steering and focusing optics at a 90° angle inside a sample holder, which allowed for a 10x10 mm rectangular spectrophotometer cells that was connected to a circulating bath for temperature

control. Additionally, the solutions were stirred during the measurements by a 3 miniature stirring plate inserted into the sample holder (Starna Cells SCS 1.11). The sample holder was placed into a metal enclosure to prevent exposure to ambient light. Both pump and probe beams were nearly collimated inside the cell with a diameter of about 2 mm. The pump beam was blocked after passing through the sample and the probe beam was directed by a system of lenses into the detector (Ocean Optics Flame-S1-XR spectrometer), which acquired spectra of the probe light. The detector was connected to a PC via USB port. The experiment was controlled by a National Instrument LabVIEW program which collected the probe light spectra, determined sample optical absorption spectra, controlled pump and probe light sources, and stored the data on the computer S3 hard drive according to the experimental protocol. Experiments were performed at 10 μM concentration unless otherwise stated.

1.3 Synthesis of DASA small molecules.

The following general experimental procedure was used for the synthesis of the DASA-small molecules used to determine the solvatochromic slopes of the DASAs present in the LCEs. 200 mg (1.05 mmol, 1 equivalent) of 4-methoxy-N-(pent-4-en-1-yl)aniline (prepared using reported literature[2]) were added to a one-dram vial and dissolved in 1 mL of dichloromethane and hexafluoroisopropanol (9:1). To this solution, 1.6 mmol (1.5 equivalents) of the acceptor (prepared using reported literature[3–5]) were added. The reactions were let stirring 3-4 hours. After this, the reactions were purified by flushing them with dichloromethane through a small silica plug to elute the starting materials. Then, the silica plug was flushed with a mixture of 10 % methanol in dichloromethane. The solution was concentrated under reduced pressure to yield the DASA products.

Synthesis of (Z)-4-((2Z,4E)-2-hydroxy-5-((4-methoxyphenyl)(pent-4-en-1-yl)amino)penta-2,4-dien-1-ylidene)-5-methyl-2-phenyl-2,4-dihydro-3H-pyrazol-3-one, (Methylpyrazolone derivative).

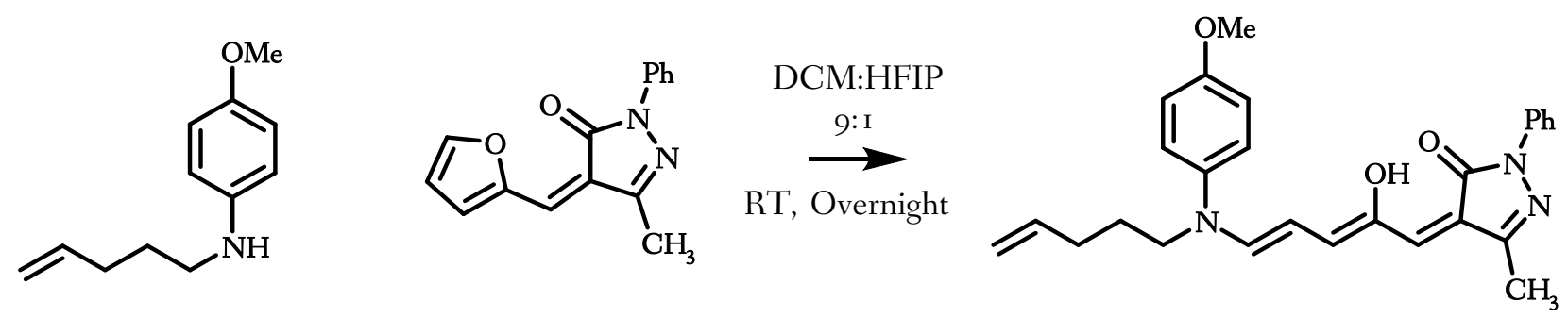

Synthesis of the Methyl-Pyrazolone derivative was done following the general procedure described above. The product was obtained as a deep blue wax. Yield 83 %, 555 mg, 1.0 mmol. ^{1}H NMR (400 MHz, CD_2Cl_2) δ 12.73 (s, 1H), 7.97 – 7.91 (m, 2H), 7.37 – 7.30 (m, 2H), 7.13 – 7.08 (m, 2H), 7.06 – 7.01 (m, 2H), 6.89 – 6.84 (m, 2H), 6.39 (s, 2H), 5.98 (s, 1H), 5.72 (ddt, *J* = 19.1, 9.6, 6.6 Hz, 1H), 5.03 – 4.94 (m, 2H), 3.76 (s, 3H), 3.68 (t, *J* = 9.3 Hz, 2H), 2.16 (s, 3H), 2.04 (q, *J* = 7.2 Hz, 2H), 1.70 (q, *J* = 7.5 Hz, 2H). ^{13}C NMR (100 MHz, CD_2Cl_2) δ 164.82, 164.41, 158.25, 150.12, 148.91, 142.77, 141.40, 139.01, 138.17, 138.02, 136.80, 136.20, 128.86, 128.68, 124.49, 121.85, 120.53, 119.41, 118.87, 118.64, 116.09, 115.00, 114.91, 114.41, 114.05, 113.65, 102.92, 77.54, 77.42, 77.22, 76.90, 55.83, 55.57, 55.51, 53.57, 44.41, 31.35, 31.13, 30.66, 28.75, 27.46, 26.53, 12.92. (ATR) FTIR,2959.6, 2931.8, 2858.2, 1725.3, 1630.9, 1595.0, 1567.8, 1521.9, 1489.3, 1443.9, 1388.2, 1369.7, 1322.6, 1279.2, 1246.3, 1208.8, 1147.2, 1111.8, 999.8, 986.0, 907.2. HR-MS (ES+) [M+H] calc: 444.22 m/z, found: 444.22 m/z.

Synthesis of 5-((2Z,4E)-2-hydroxy-5-((4-methoxyphenyl)(pent-4-en-1-yl)amino)penta-2,4-dien-1-ylidene)-2,2-dimethyl-1,3-dioxane-4,6-dione, (Meldrum's derivative).

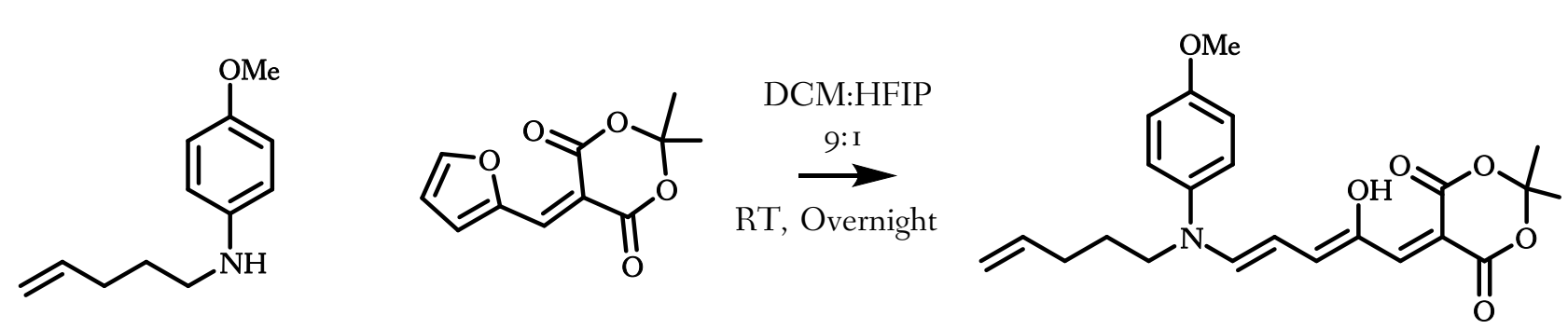

Synthesis of the Meldrum derivative was done following the general procedure described above. The product was obtained as a deep blue wax. Yield 74 % 435 mg, 1.0 mmol. ^{1}H and ^{13}C NMR spectra matched with the previously reported spectra.[3]

Synthesis of 5-((2Z,4E)-2-hydroxy-5-((4-methoxyphenyl)(pent-4-en-1-yl)amino)penta-2,4-dien-1-ylidene)-1,3-dimethylpyrimidine-2,4,6(1H,3H,5H)-trione, (Barbituric derivative).

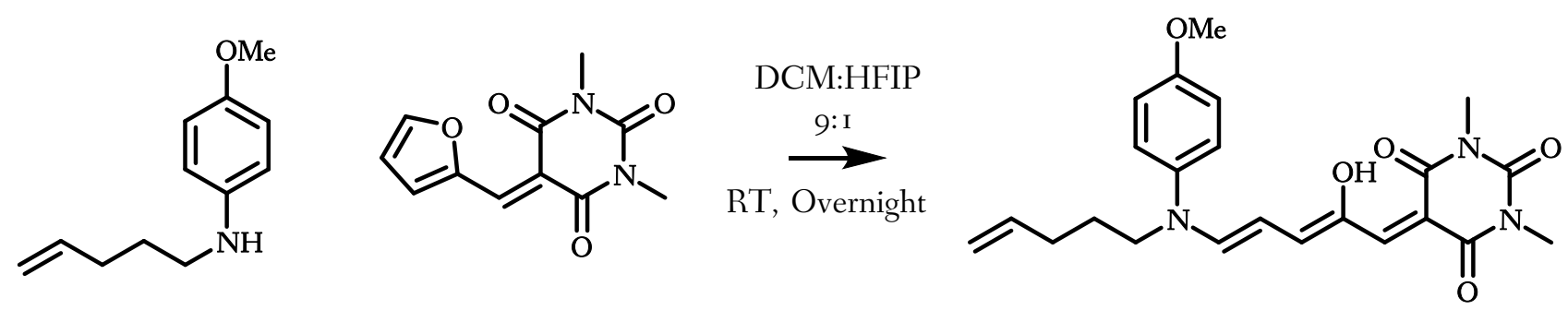

Synthesis of the Barbituric derivative was done following the general procedure described above. The product was obtained as a deep violet wax. Yield 67 %, 393 mg, 0.8 mmol. ^1H and ^{13}C NMR spectra matched with the previously reported spectra.[3]

Synthesis of (Z)-4-((2Z,4E)-2-hydroxy-5-((4-methoxyphenyl)(pent-4-en-1-yl)amino)penta-2,4-dien-1-ylidene)-3-methylisoxazol-5(4H)-one, (Methyloxazolone derivative).

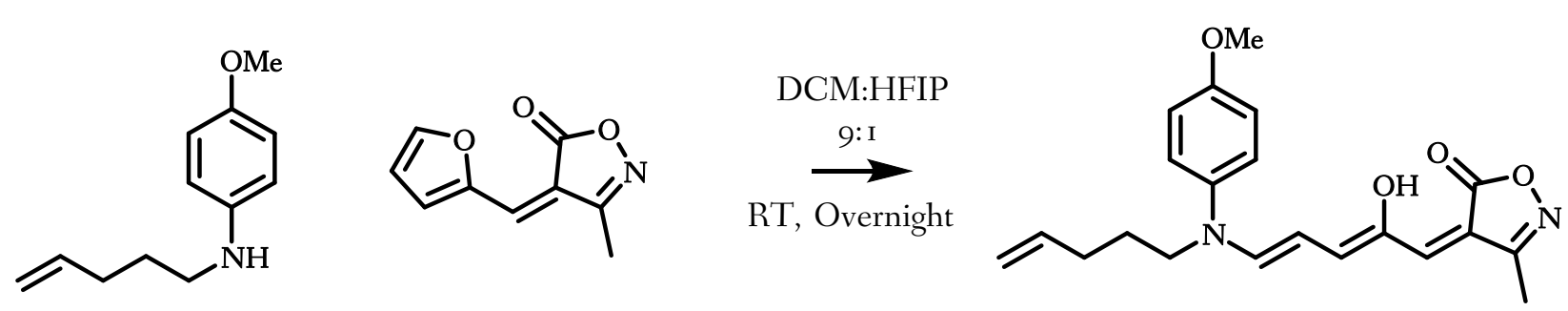

Synthesis of the Oxazolone derivative was done following the general procedure described above. The product was obtained as a deep violet wax. Yield 92%, 540 mg, 1.2 mmol. ^1H NMR of the open form which exists on the open form (95%) (400 MHz, CD_2Cl_2) δ 7.39 – 7.31 (m, 1H), 7.13 (d, *J* = 8.3 Hz, 2H), 6.93 (d, *J* = 8.4 Hz, 2H), 6.86 – 6.66 (m, 0H), 6.54 (d, *J* = 12.2 Hz, 1H), 6.24 (s, 1H), 5.73 (d, *J* = 9.1 Hz, 1H), 5.06 – 4.93 (m, 2H), 3.78 (d, *J* = 21.0 Hz, 5H), 2.08 (d, *J* = 9.1 Hz, 4H), 1.74 (s, 1H). ^{13}C NMR (100 MHz, CD_2Cl_2) δ 175.75, 160.71, 152.70, 149.69, 148.40, 145.20, 137.96, 136.55, 134.64, 132.01, 127.28, 126.11, 123.54, 116.30, 115.18, 114.93, 114.49, 103.67, 77.44, 77.33, 77.12, 76.81, 55.81, 55.65, 50.58, 31.23, 30.60, 27.41, 26.21, 11.36, 11.17. (ATR) FTIR, 2957.9, 2931.5, 2860.2, 1729.0, 1630.1, 1536.3, 1491.5, 1446.9, 1381.9, 1246.5, 1208.6, 1127.1, 985.4, 944.1, 896.8. HR-MS (ES+) [M+H] calc: 369.17 m/z, found: 369.18 m/z.

Synthesis of (Z)-1-butyl-5-((2Z,4E)-2-hydroxy-5-((4-methoxyphenyl)(pent-4-en-1-yl)amino)penta-2,4-dien-1-ylidene)-4-methyl-2,6-dioxo-1,2,5,6-tetrahydropyridine-3-carbonitrile (Hydropyridine derivative).

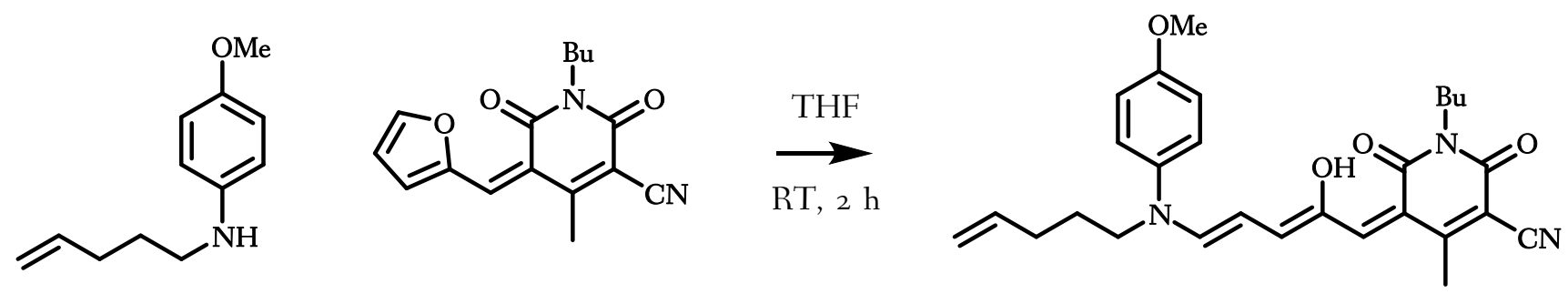

Synthesis of the Hydropyridine derivative was done following modification of general procedure substituting THF as solvent and reducing the reaction duration to 2 hours. The product was obtained as a bright blue powder. Yield 22 %, 120 mg, 0.3 mmol. Synthesized DASA is unstable in open form. The existence of open form is confirmed visually by its bright color. The open form is unstable in silica and trituration is not able to fully remove the acceptor.

1.4 Kinetics of DASA-Fication in Linear Model Polymer.

Synthesis of the donor-functionalized linear model polymer: Polymethylhydrosiloxane (PHMS, 72.2 mg, 1.2 mmol Si–H), the mesogen (4-methoxyphenyl 4-(but-3-en-1-yloxy)benzoate (357.8 mg, 1.199 mmol), and the donor 4-methoxy-N-(pent-4-en-1-yl)aniline (0.00084 mmol, delivered as 7 μL of an 18.4 mg/mL stock solution) were combined in 1.5 mL of toluene in the presence of 10 μL of a 1 wt% (1,5-cyclooctadiene)-platinum(II) dichloride catalyst solution. The reaction was stirred continuously under N₂ at 70 °C for 1 h. This feed ratio corresponds to a donor loading of 0.07 mol% relative to total Si–H content, with the mesogen and donor together stoichiometrically matching the Si–H functionality of PHMS. Following the reaction, toluene was removed under reduced pressure, and the resulting donor-functionalized linear polymer was redissolved in 1 mL of dichloromethane (DCM) to generate a stock solution.

UV-Vis kinetics measurements: All kinetics measurements were performed in solution. Aliquots of the linear polymer stock solution (100 μL) were added to 2 mL of a 25 mg/mL solution of each acceptor (M-Pyra, Mel, Barb, M-Oxa, or HDP) in 9:1 dichloromethane:hexafluoroisopropanol (DCM:HFIP), and the evolution of the characteristic DASA absorption peak was monitored by UV-Vis absorption spectroscopy, with absorbance recorded every 10 s over 60 min. Each corresponding 2 mL acceptor-only solution (without added polymer) was used as the baseline blank for its respective measurement; because the acceptor and the resulting DASA chromophore are spectrally well separated, this blanking approach allows the emerging DASA absorbance to be tracked without interference from the residual, unreacted acceptor. For Barb, the slowest-reacting acceptor within the initial 60 min window, the measurement was extended to 16 h, with absorbance recorded every 5 min, to determine whether the reaction reached maximum conversion.

1.5 Synthesis of DASA LCE films by Finkelmann's method.

The required amounts of mesogen (4-methoxyphenyl 4-(but-3-en-1-yloxy)benzoate, mass: 286.3 mg), crosslinker (1,4-bis(undec-10-en-1-yloxy)benzene, mass: 49.8 mg), polymer (polyhydromethylsiloxane, DP = 180, mass: 72.2 mg) and donor (4-methoxy-N-(pent-4-en-1-yl)aniline, 7 μL out of 18.4 mg/mL stock solution) are measured by mass (except the donor) and added to a scintillation vial. To this vial, 1.5 mL of toluene are added and the crosslinker, polymer and mesogen are dissolved. Then, a volume of the stock solution of donor is added and mixed with the other monomer motifs. The polymerization of the LCE is catalyzed with (1,5-cyclooctadiene)-platinum(II) dichloride (10 μL out of 10 mg/mL stock solution) in the amounts shown in **Table S1**. The catalyst is added and mixed with the other monomers right before pouring the mixture into the pre-heated centrifuge. After 60 min at 70 °C, the setup is cooled down at room temperature over 2 hours and the lightly crosslinked sample is taken out of the centrifuge and removed from the Teflon holder. The samples are subsequently allowed to air dry for approximately 15 min before hanging the samples vertically. Weight (2 g) is hung from the film and the sample are

allowed to react overnight at room temperature. Of note, the weight is placed on the film after the edges start appearing white. The aligned and cross-linked films are then added to a scintillation vial with toluene (10 mL) for 30 min and then to scintillation vial with dichloromethane (DCM, 10 mL) for 60 min, three times, to remove of the Platinum catalyst. Upon removal of the platinum catalyst, the film is added to another scintillation vial containing a stock solution of the acceptors (about 25 mg / mL with 10% HFIP) in DCM (10 mL). The film is allowed to soak overnight, upon which it becomes dark blue. Subsequently the films are soaked in a DCM solution (10 mL) (two times for 1 hr each) and then soaked in a toluene solution (10 mL, one time for one hr). The resulting films are dried at room temperature and used without further purification.

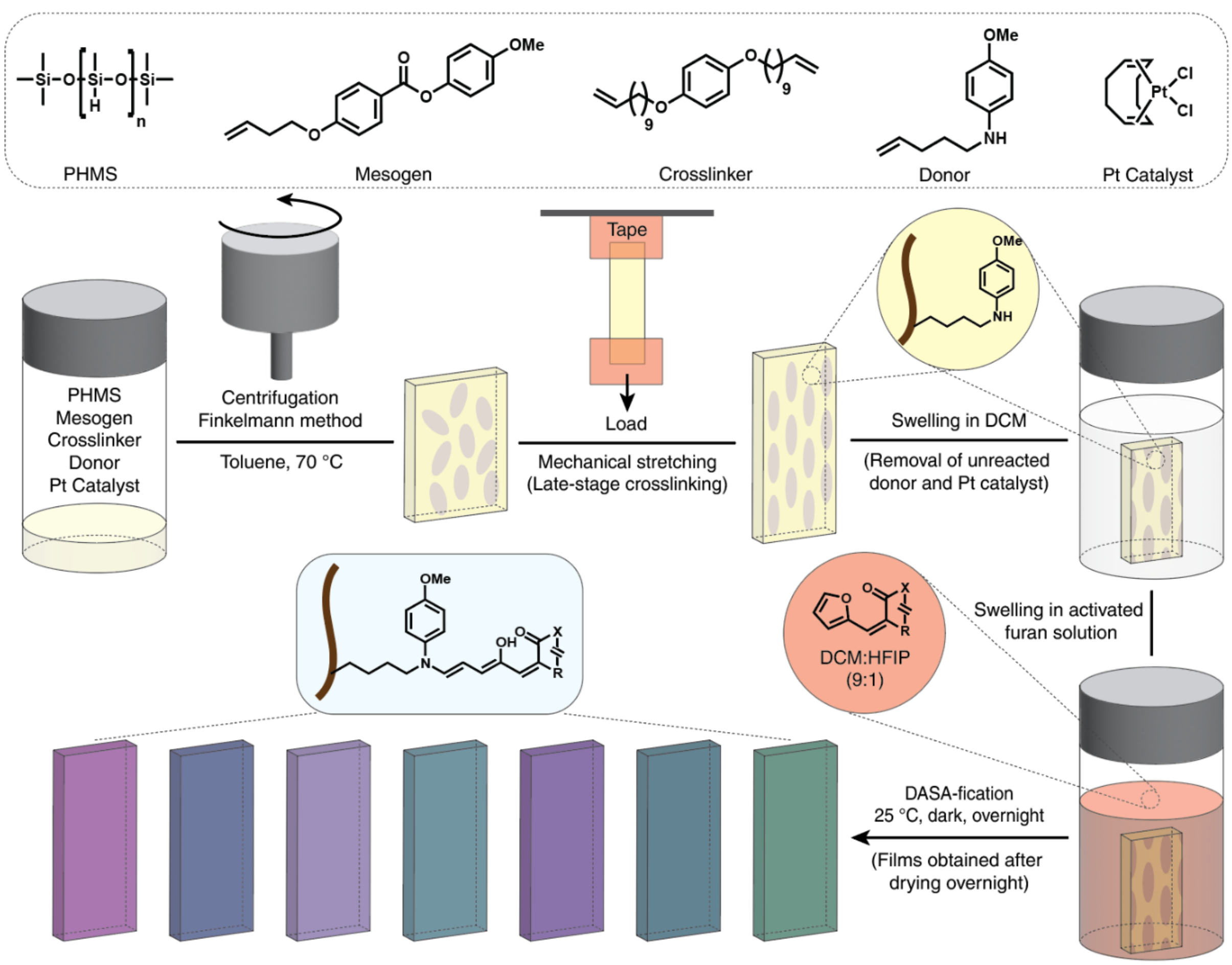

Scheme S1. Late-stage functionalization used to introduce DASA in polymethylsiloxane LCEs.

1.6 Fabrication and operation of HDP-LCE bilayer actuators.

HDP-LCE films (10 mm × 3 mm × 0.5 mm) were laminated with a strip of optically transparent adhesive tape cut to identical lateral dimensions. The tape was applied to the top surface of the LCE with uniform manual pressure to ensure conformal contact and eliminate interfacial air gaps. The LCE layer was oriented with its nematic director aligned along the long axis of the strip (10 mm direction) to maximize uniaxial contraction along the primary bending axis. For jumping experiments, a high-loading variant was prepared identically using HDP-LCE synthesized with a tenfold increase in DASA concentration (0.7 mol% relative to the standard 0.07 mol%), with all other fabrication parameters unchanged. Bilayer actuators were placed LCE-side down on a flat or grooved substrate and illuminated from below using a broadband visible light source (300 $mW.cm^{-2}$). The light source was positioned beneath the substrate such that the LCE layer was proximal to the illumination. Time-lapse imaging was performed using a digital camera positioned perpendicular to the plane of actuation. For crawling experiments, a grooved substrate was used to impart directional asymmetry to the actuation cycle. Locomotion velocity was estimated by tracking the displacement of the actuator centroid across sequential frames over a recorded interval of 0–12 s, yielding an average velocity of approximately 0.2 cm s⁻^{1}.

2. Results

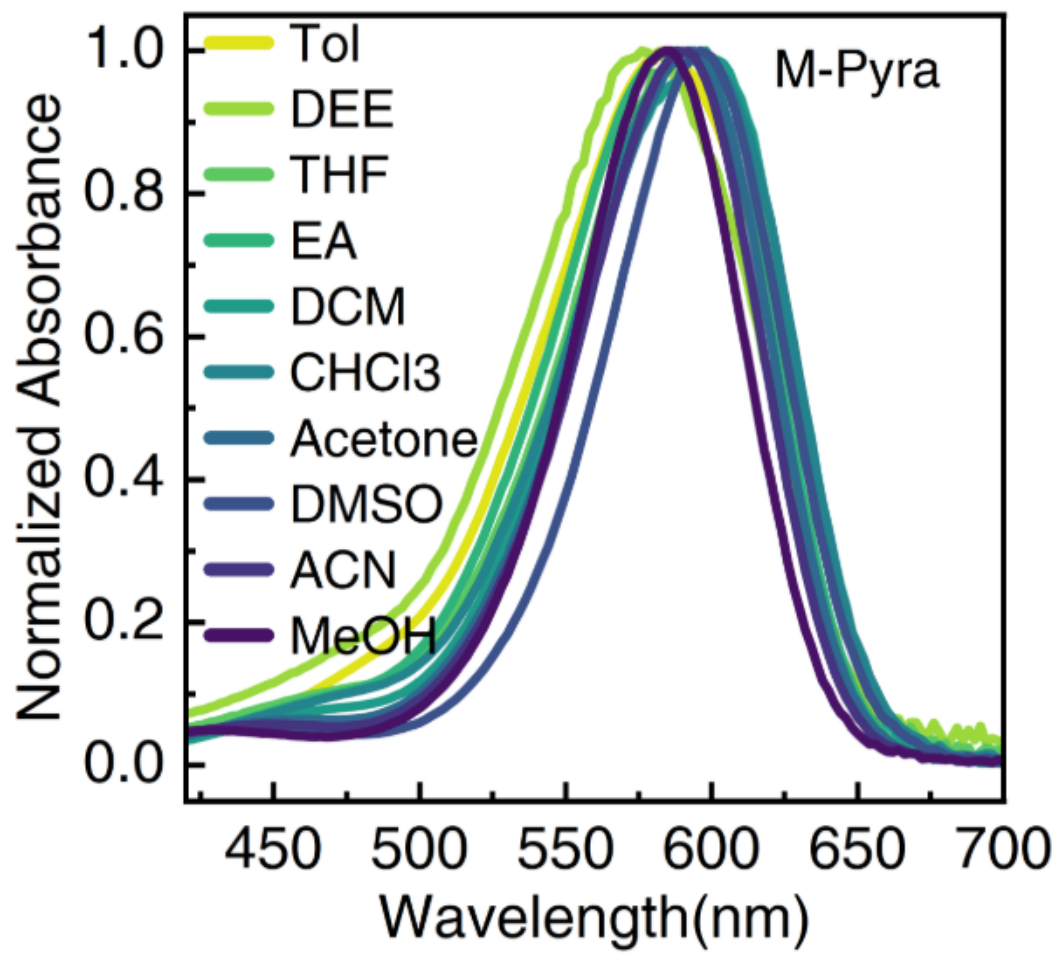

Figure S1. Spectral bands of the Methyl-pyrazolone derivative DASA-small molecule model system in ten different solvents.

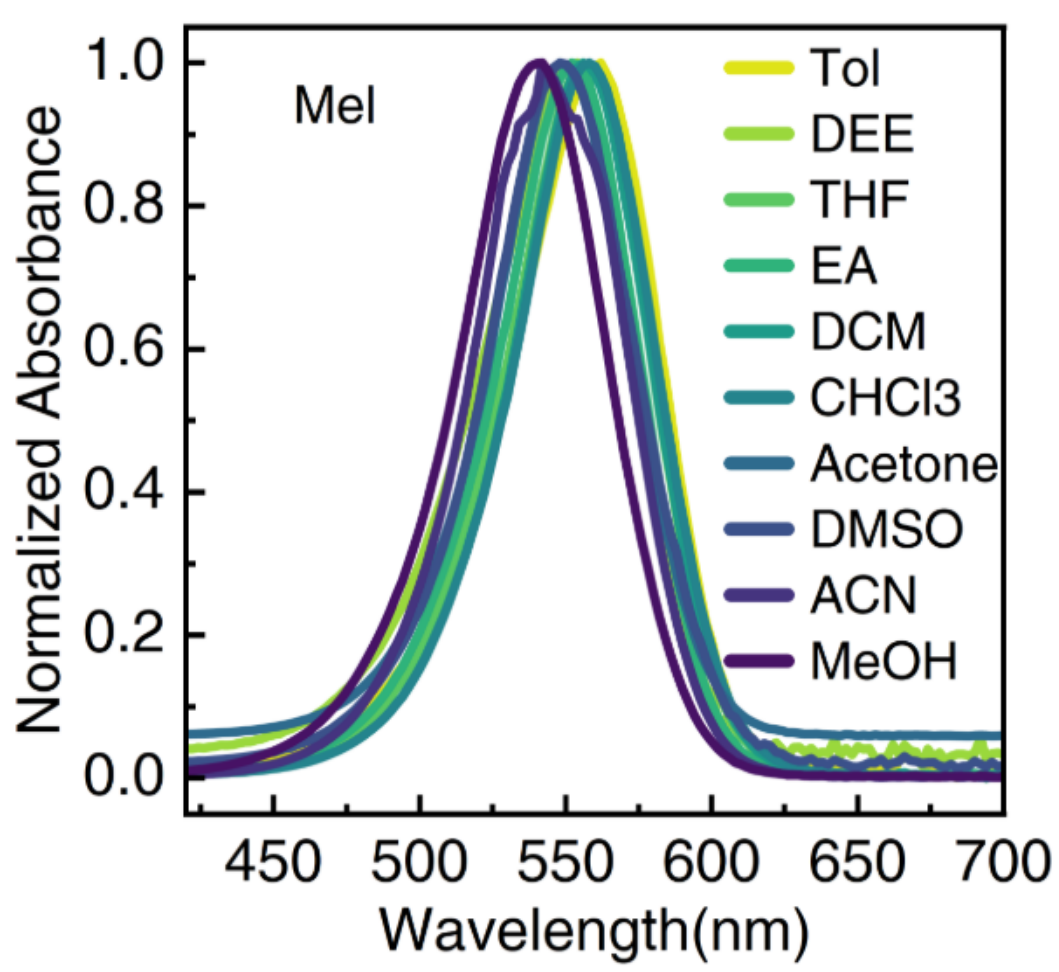

Figure S2. Spectral bands of the Meldrum acid derivative DASA-small molecule model system in ten different solvents.

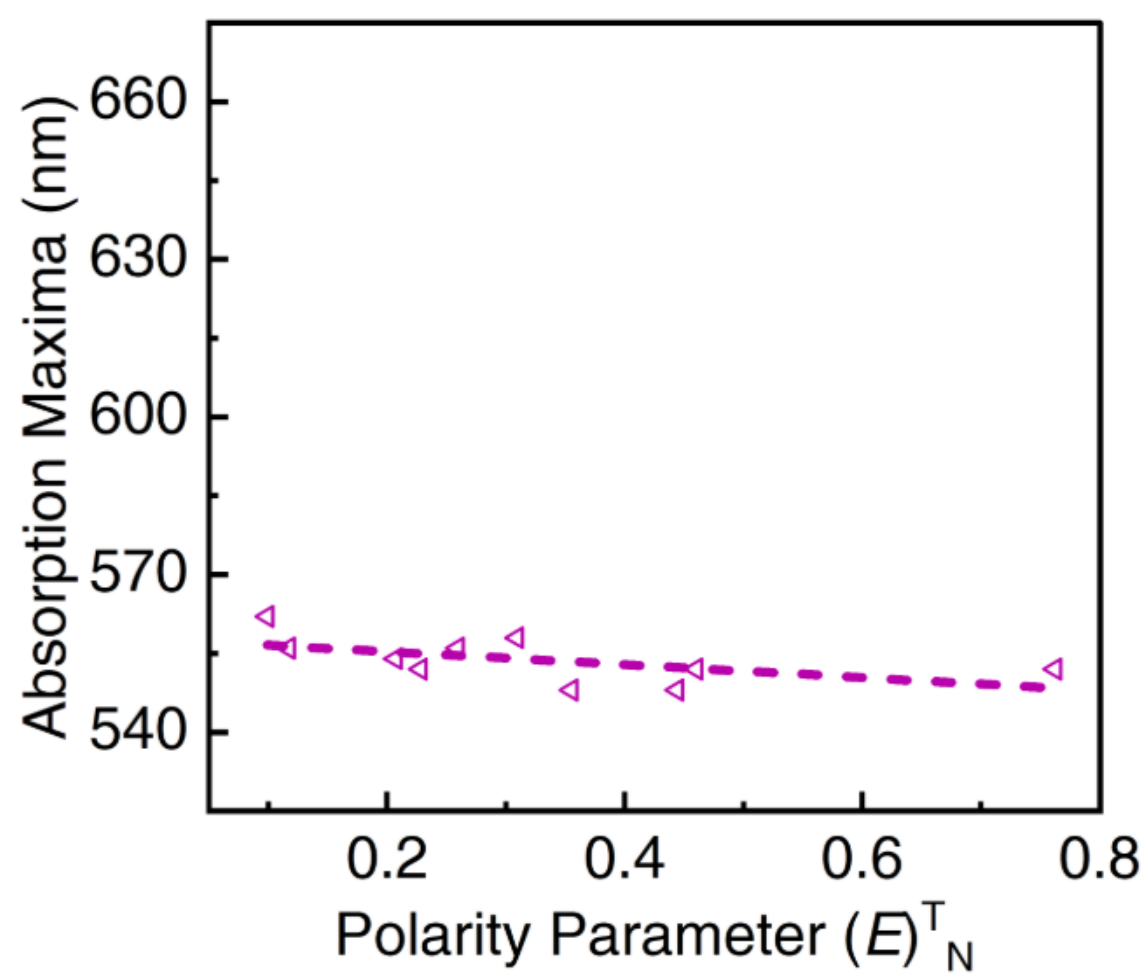

Figure S3. Solvatochromic slope of the Meldrum's acid derivative DASA-small molecule model system obtain by the linear fit of the absorbance maxima as a function of the solvent polarity.

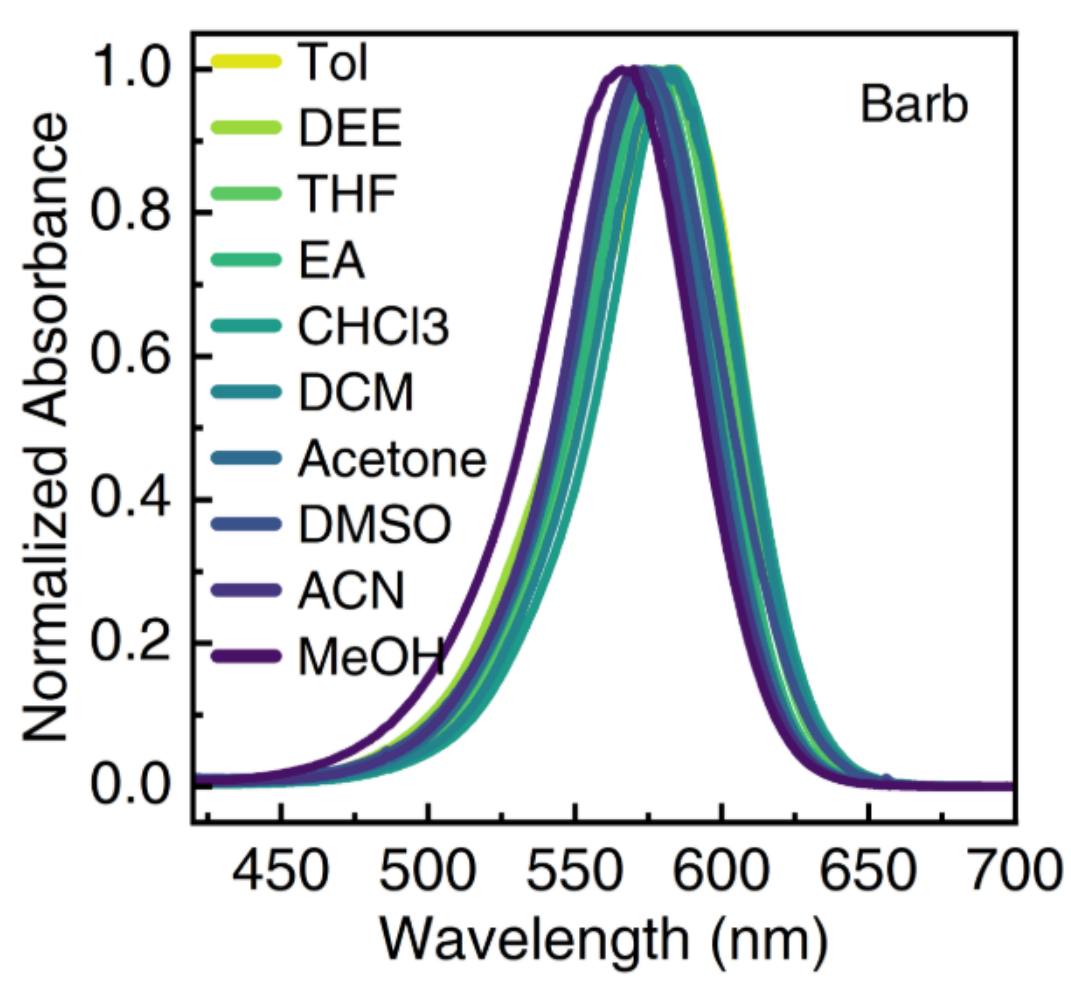

Figure S4. Spectral bands of the Barbituric acid derivative DASA-small molecule model system in ten different solvents.

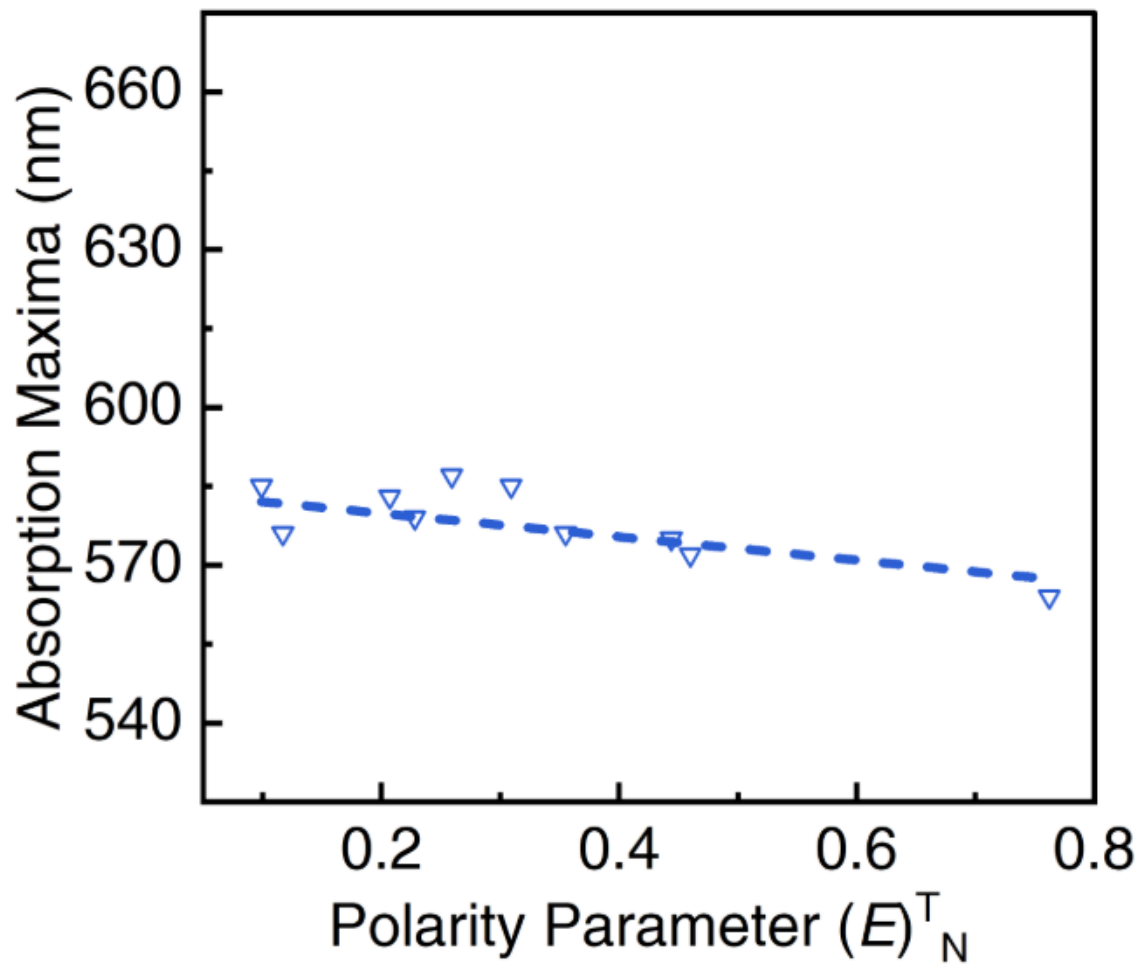

Figure S1. Solvatochromic slope of the Barbituric acid derivative DASA-small molecule model system obtain by the linear fit of the absorbance maxima as a function of the solvent polarity.

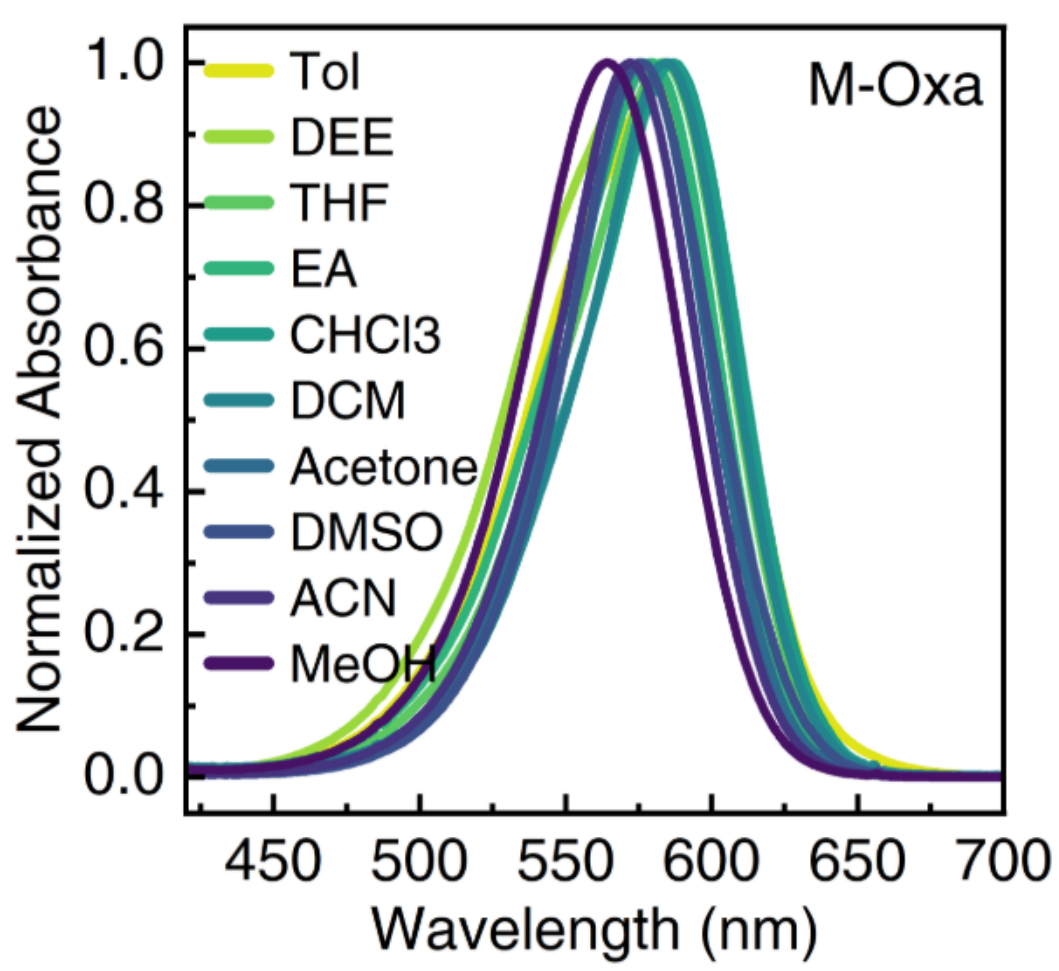

Figure S6. Spectral bands of the Methyl-oxazolone derivative DASA-small molecule model system in ten different solvents.

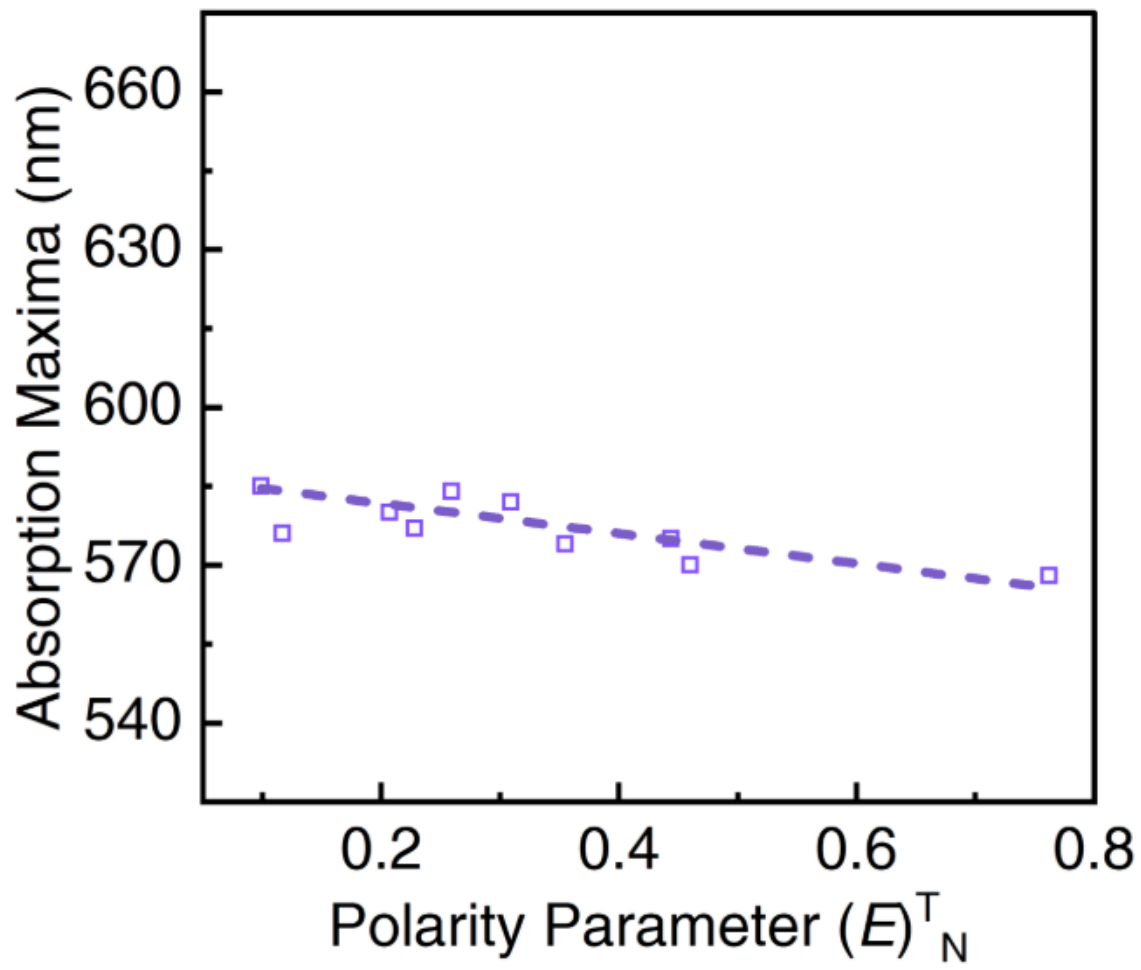

Figure S7. Solvatochromic slope of the Methyl-oxazolone derivative DASA-small molecule model system obtain by the linear fit of the absorbance maxima as a function of the solvent polarity.

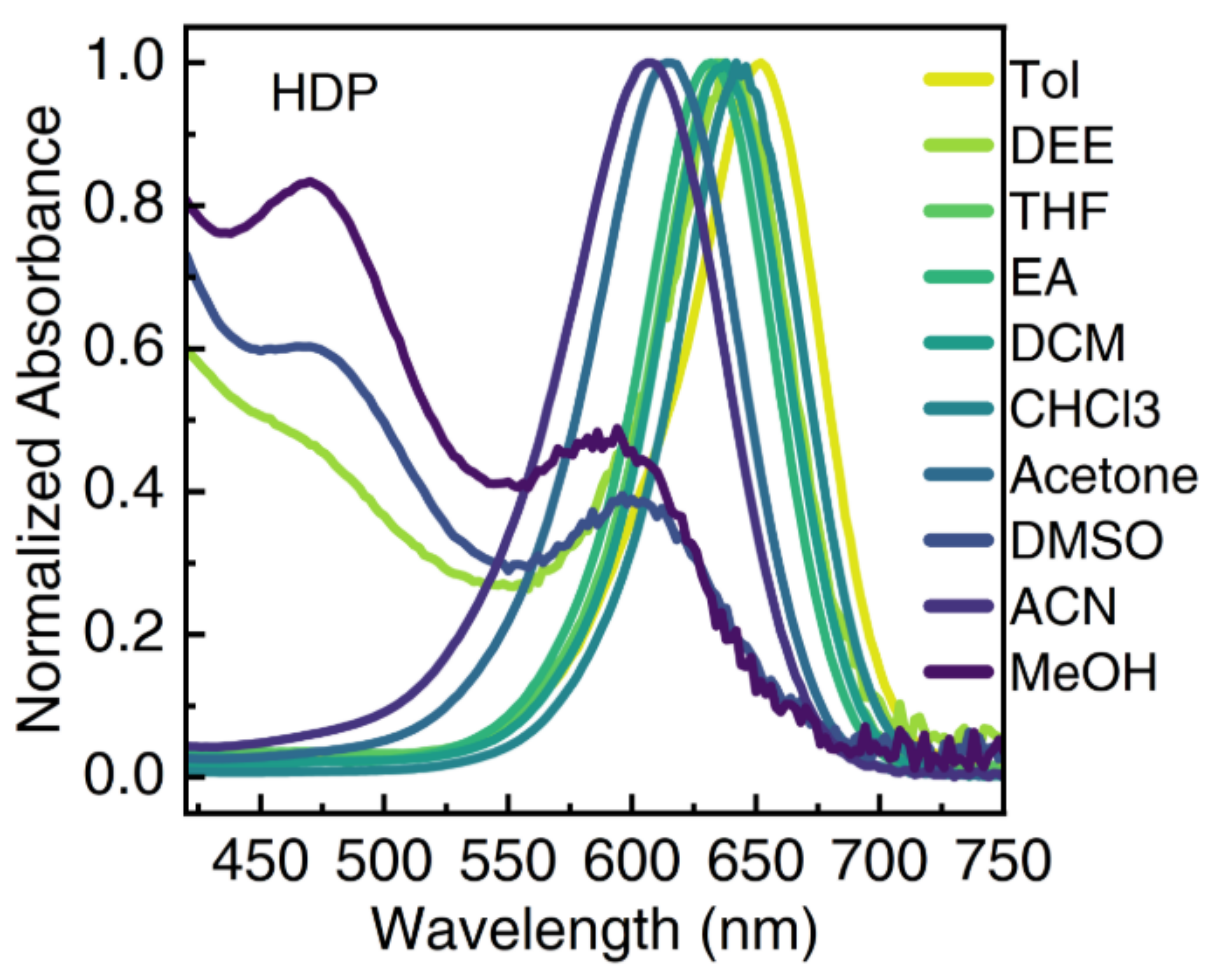

Figure S8. Spectral bands of the Hydroxypyridone derivative DASA-small molecule model system in ten different solvents.

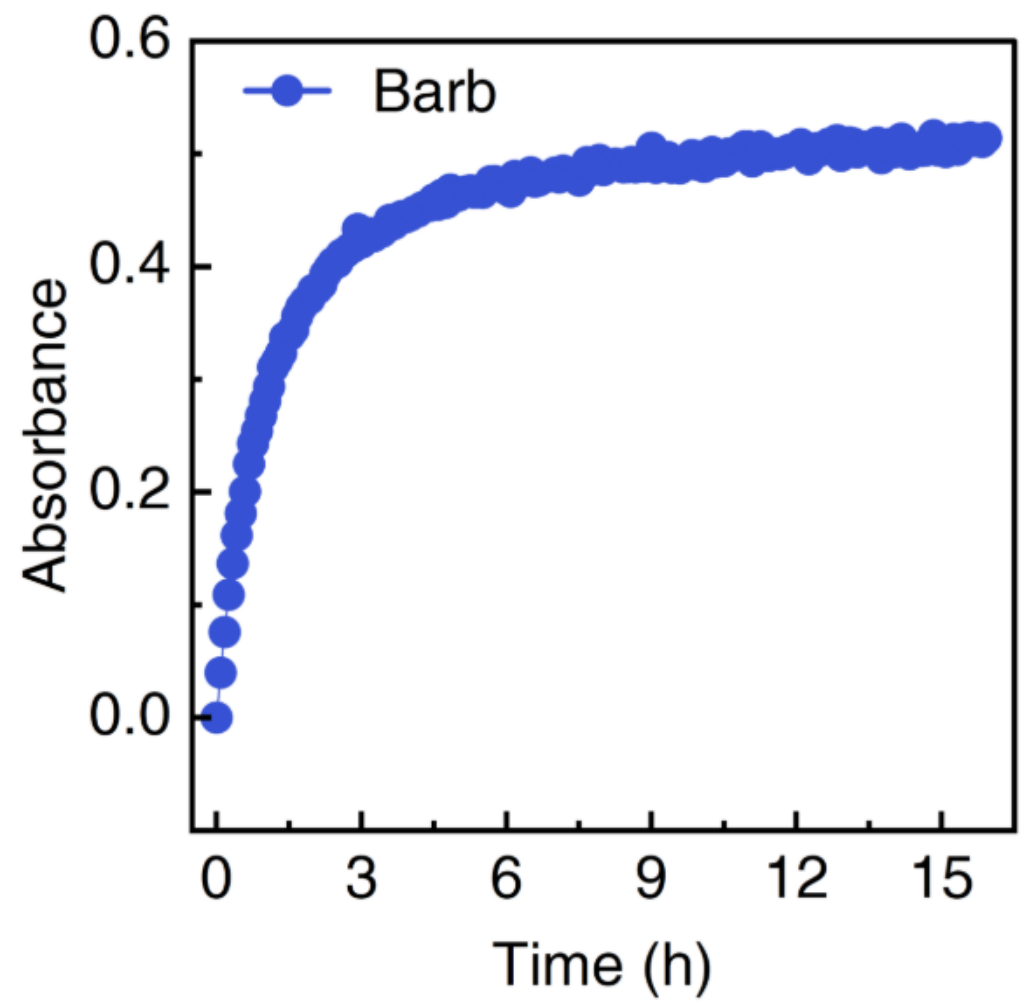

Figure S9. Kinetic evolution of Barb-DASA inside non-crosslinked linear polyhydromethylsiloxane (mesogen and donor amine functionalized)

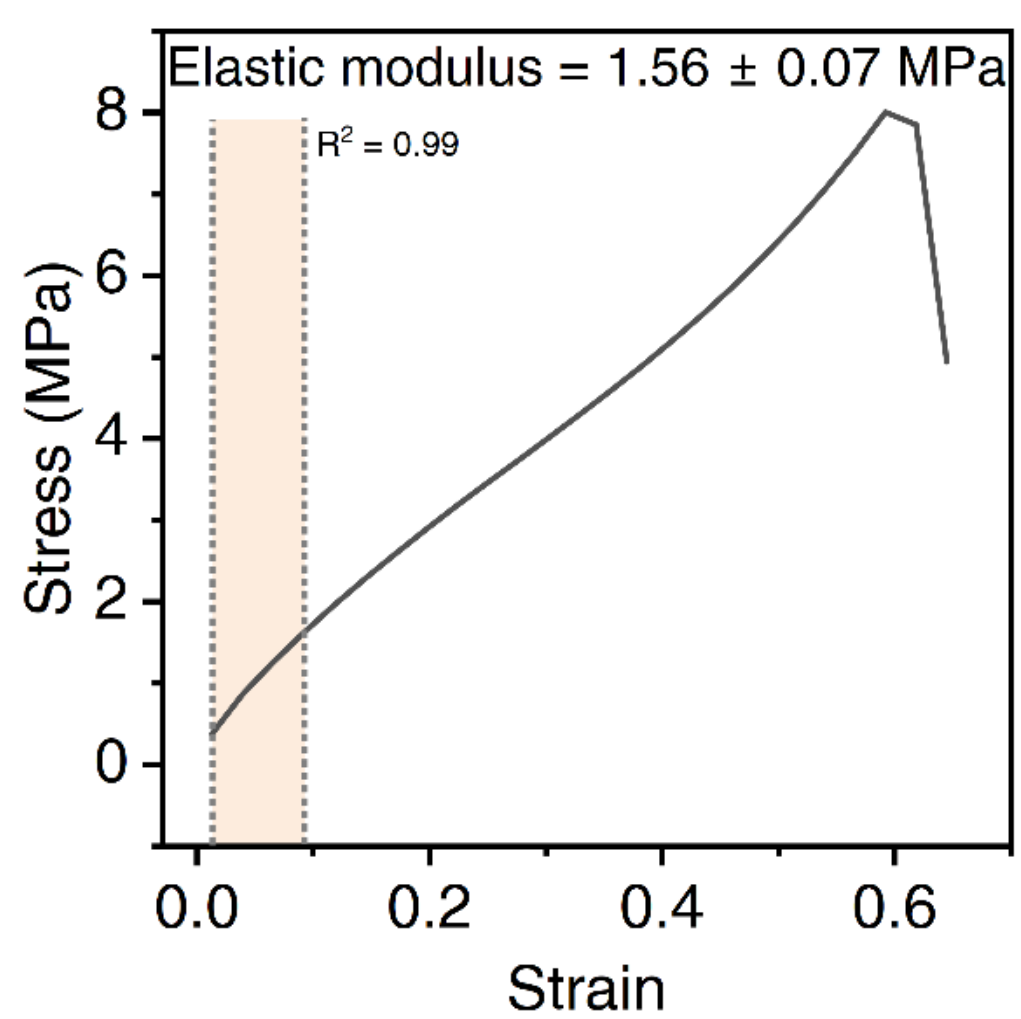

Figure S10. Tensile testing of Donor-LCE (before DASA-functionalization)

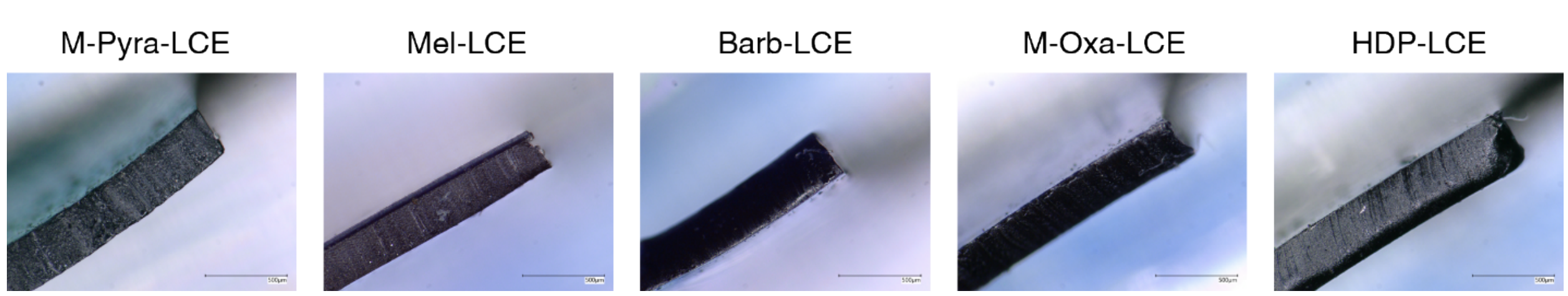

Figure S11. Bright field images of the cross-section of DASA-LCEs under microscope.

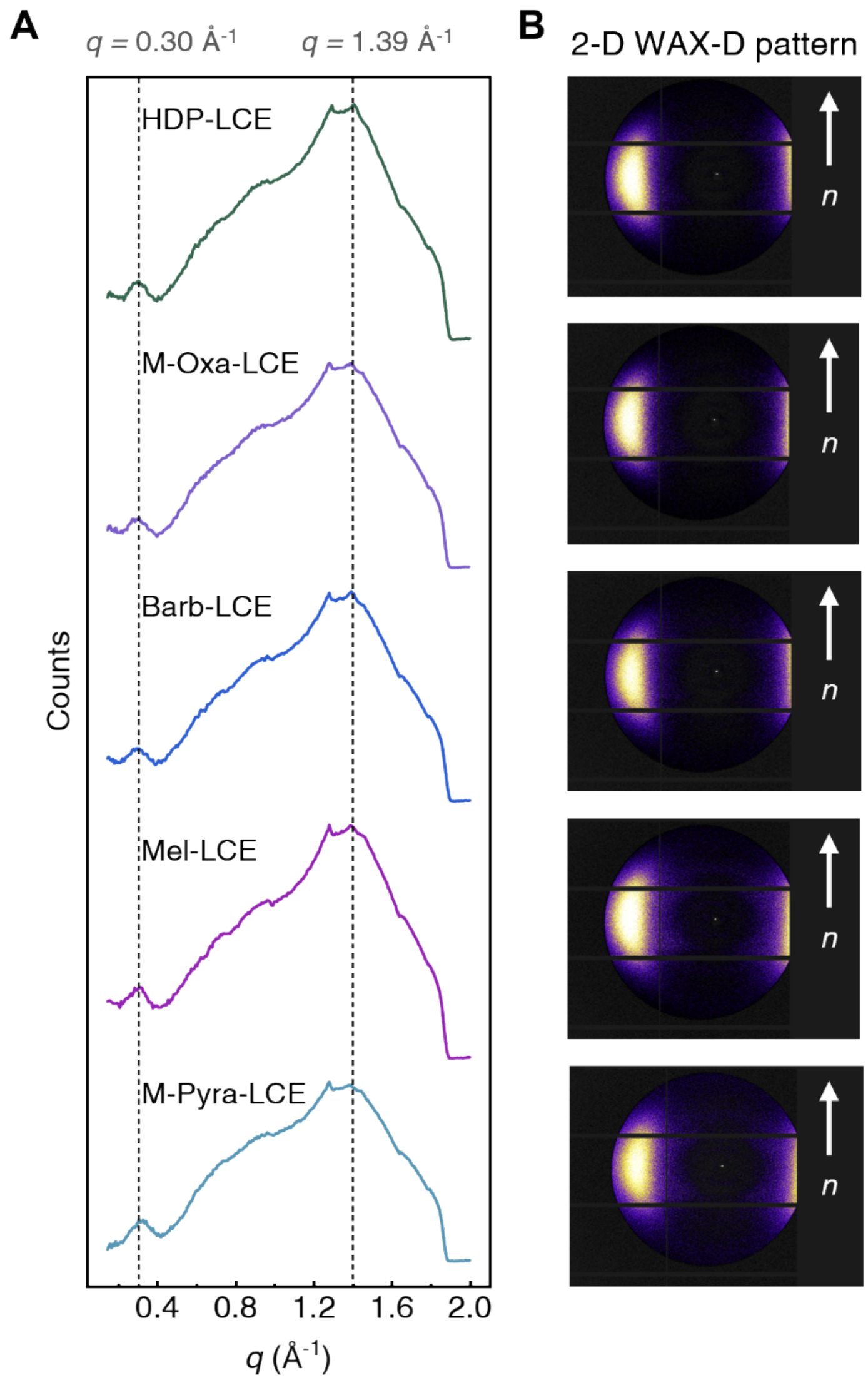

Figure S12. 1D and 2D WAX-D pattern of the DASA-LCEs.

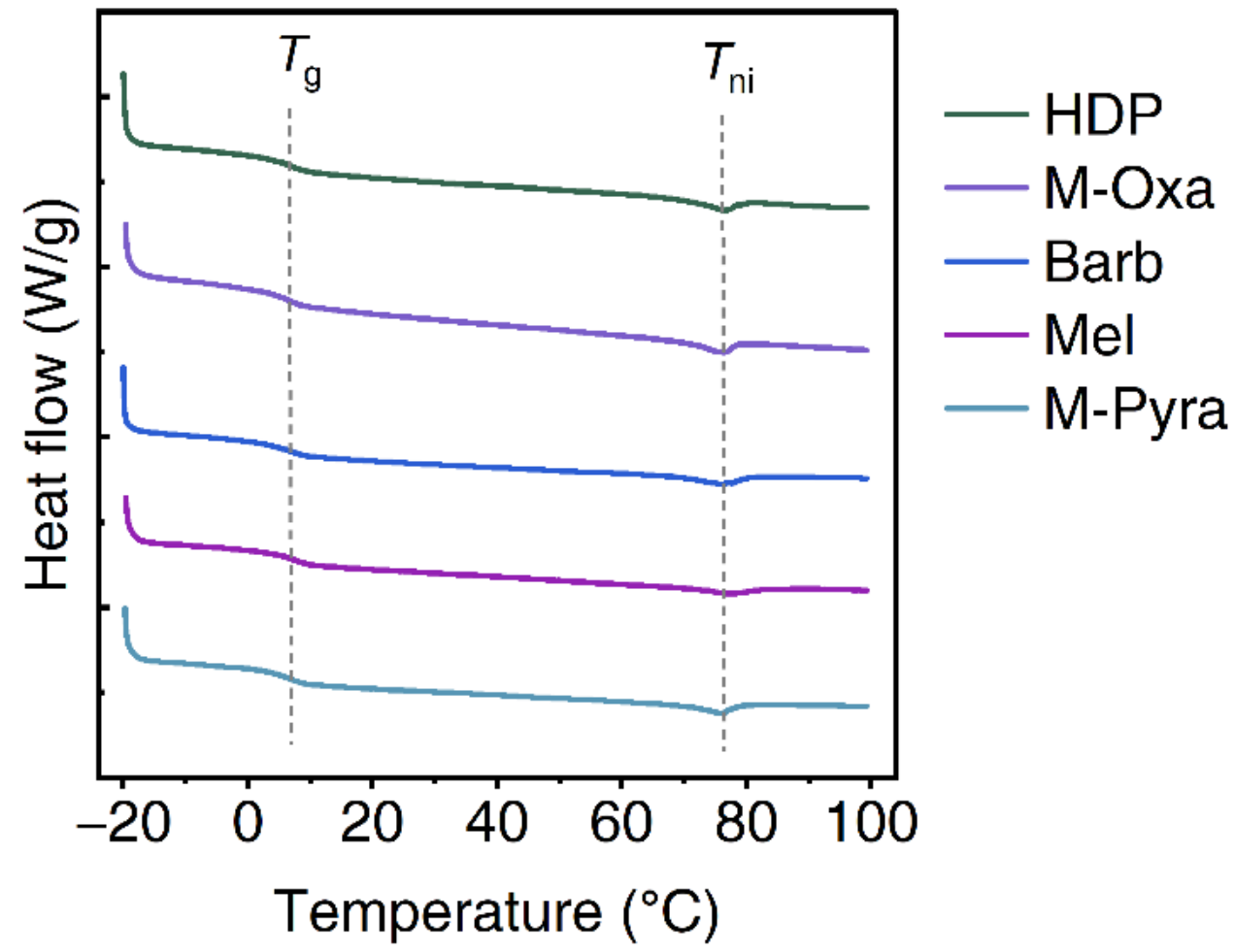

Figure S13. Differential scanning calorimetry (DSC) of five DASA-LCE films. The glass transition and nematic-isotropic transitions are annotated.

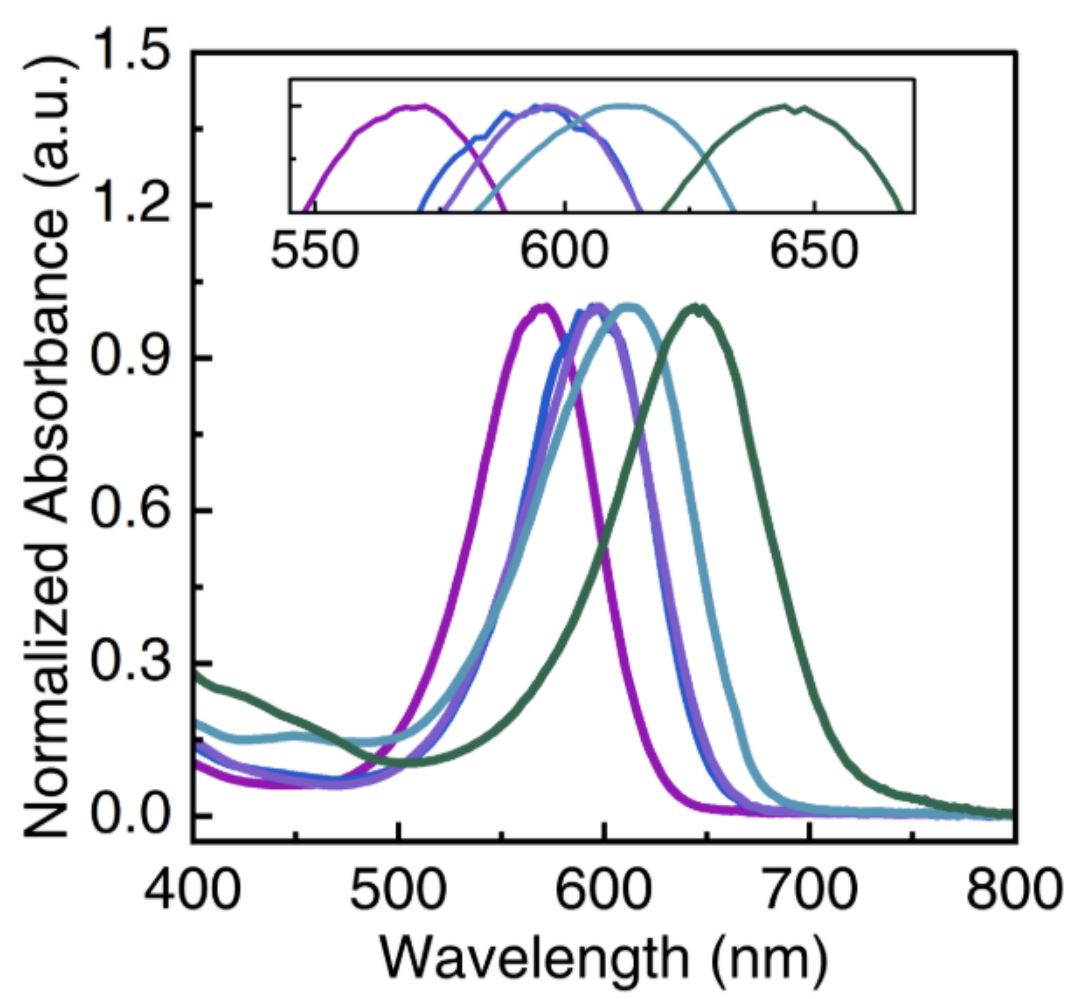

Figure S14. Absorption spectral bands of five DASA-LCE films.

Table S1. Open form absorption maxima of five DASA-LCE films.

DASA-LCE	M-Pyra	Mel	Barb	M-Oxa	HDP
λ_{max} (nm)	614	569	596	597	645

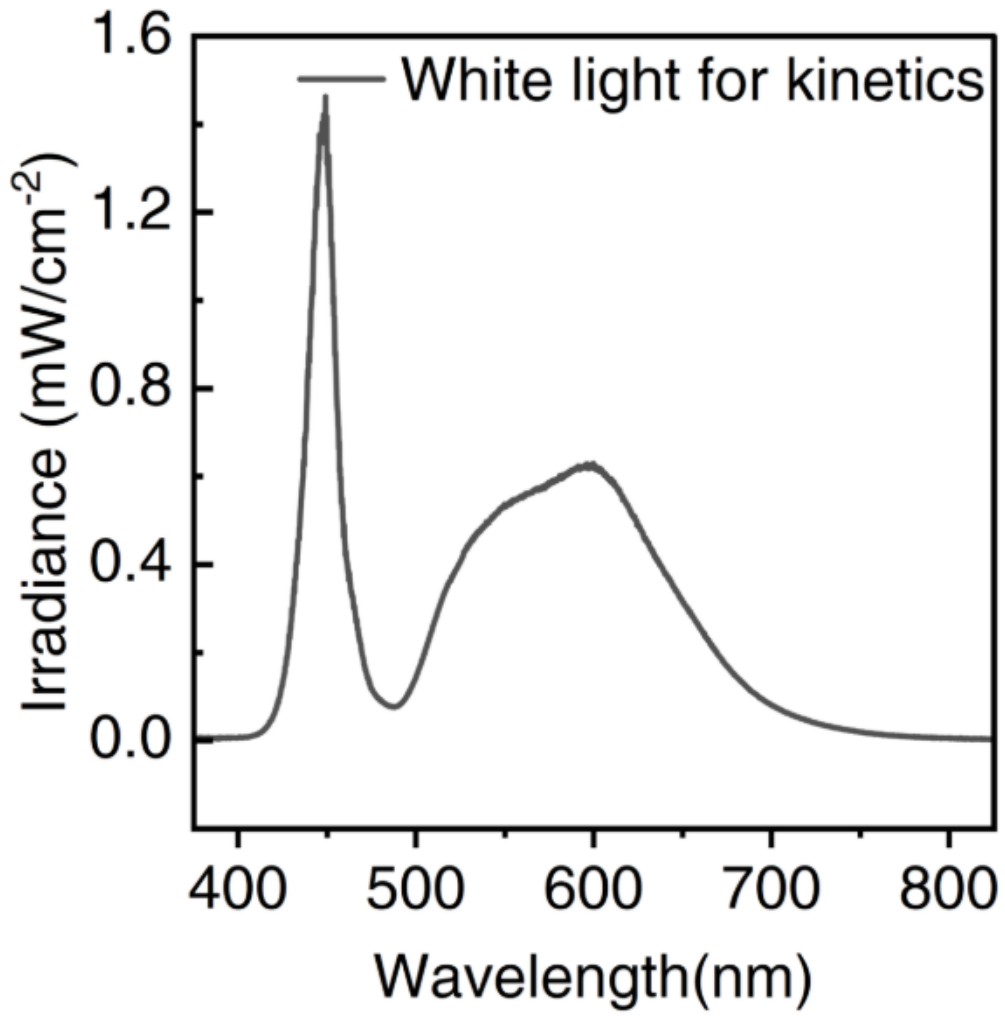

Figure S15. Spectral profile of the white light used for photoswitching kinetics studies for DASA-LCEs.

Determination of Photoswitching Rate Constants, Activation Free Energies, and Thermal Half-Lives

The photoswitching kinetics of DASA–LCE samples were monitored by tracking the time-dependent change in absorbance at the characteristic open-form absorption maximum ($\square_{max}$) under constant visible light irradiation. Upon irradiation, the absorbance decayed as the open-chain triene isomer underwent conrotatory 4π electrocyclic ring closure to the colorless cyclopentenone product.

The time-resolved absorbance traces were fitted to a single-exponential decay of the form:

$$A(t) = A_0 \, e^{-k_{PTSS} \, t}$$

where $A(t)$ is the absorbance at time t, A_0 is the pre-exponential factor corresponding to the initial absorbance at $t = 0$, and k_{PTSS} is the first-order photoswitching rate constant in units of $s^{\square 1}$. The fits for M-Pyra and Mel-LCEs were done till the early onset of PTSS. Rate constants were subsequently converted to $min^{\square 1}$ by multiplication by a factor of 60.

The thermal half-life ($t_{1/2,dark}$), defined as the time required for 50% of the open-form population to thermally revert to the closed cyclopentenone form under dark conditions, was calculated as:

$$t_{1/2,dark} = \frac{\ln 2}{k_{PTSS}} = \frac{0.6931}{k_{PTSS}}$$

The activation free energy ($\square G^{\ddagger}$) for the electrocyclic ring-closure step was determined from k_{PTSS} using the Eyring equation from transition state theory:

$$\square G^{\ddagger} = RT\ln\square\left(\frac{k_B T}{h \, \square \, k_{PSS}}\right)$$

where R is the universal gas constant (1.987 cal mol$\square^{1}$ K$\square^{1}$), T is the absolute temperature (298 K), k_B is the Boltzmann constant (1.381 × 10$\square^{23}$ J K$\square^{1}$), and h is Planck's constant (6.626 × 10$\square^{3}\square$ J s). At 298 K, $k_B T/h = 6.212 \times 10^{12}$ s$\square^{1}$ and $RT\ln\square 10 = 1.364$ kcal mol$\square^{1}$, such that the expression simplifies to:

$$\square G^{\ddagger} = 1.364 \times (16.793 - \log_{\square_{10}}(k_{PTSS}/s^{-1}))\text{kcal mol}^{-1}$$

All parameters were extracted at room temperature (298 K). The computed values of k_{PTSS}, $\log_{\square_{10}}(k_{PTSS})$, $\square G^{\ddagger}$, and $t_{1/2,dark}$ for each DASA–LCE system are summarized in Table S1.

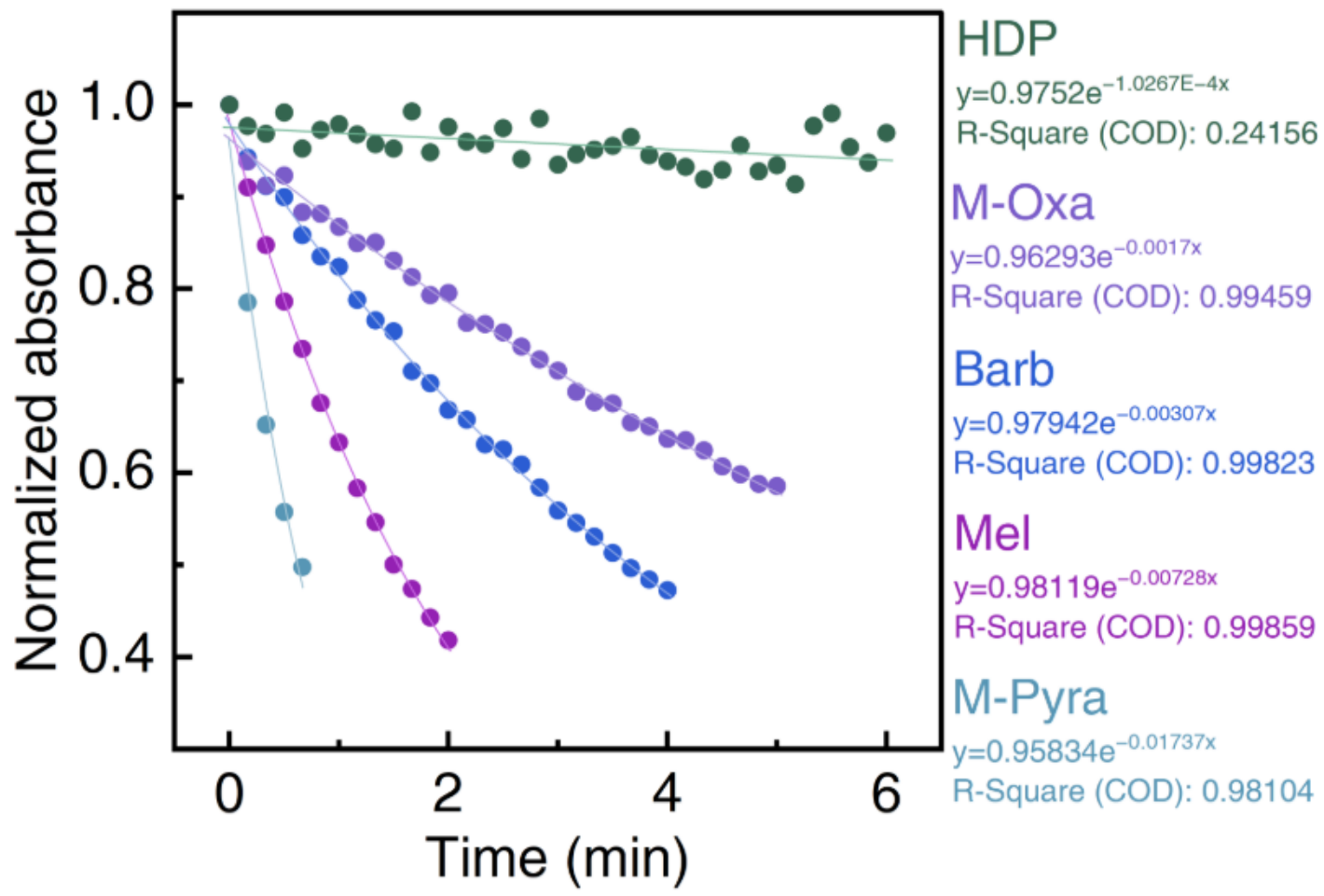

Figure S16. Photo-fading kinetic profiles of five DASA-LCE films and their first order exponential decay fit equations.

Table S2. Photo-fading rate constants, activation free energies, and thermal half-lives for DASA–LCE systems at 298 K.

**DASA**	**k (×10⁻⁴ s⁻^{1})**	**k (×10⁻^{2} min⁻^{1})**	**log₁₀(k / ×10⁻^{2} min⁻^{1})**	**ΔG‡ (kcal mol⁻^{1})**	**t₁/₂,dark (min)**
M-Pyra DASA	173.7	104.22	2.018	19.851	0.665
Mel DASA	72.8	43.68	1.6403	20.366	1.587
Barb DASA	30.7	18.42	1.2653	20.877	3.763
M-Oxa DASA	17.0	10.2	1.0086	21.227	6.796
HDP DASA	1.0	0.62	-0.2104	22.89	

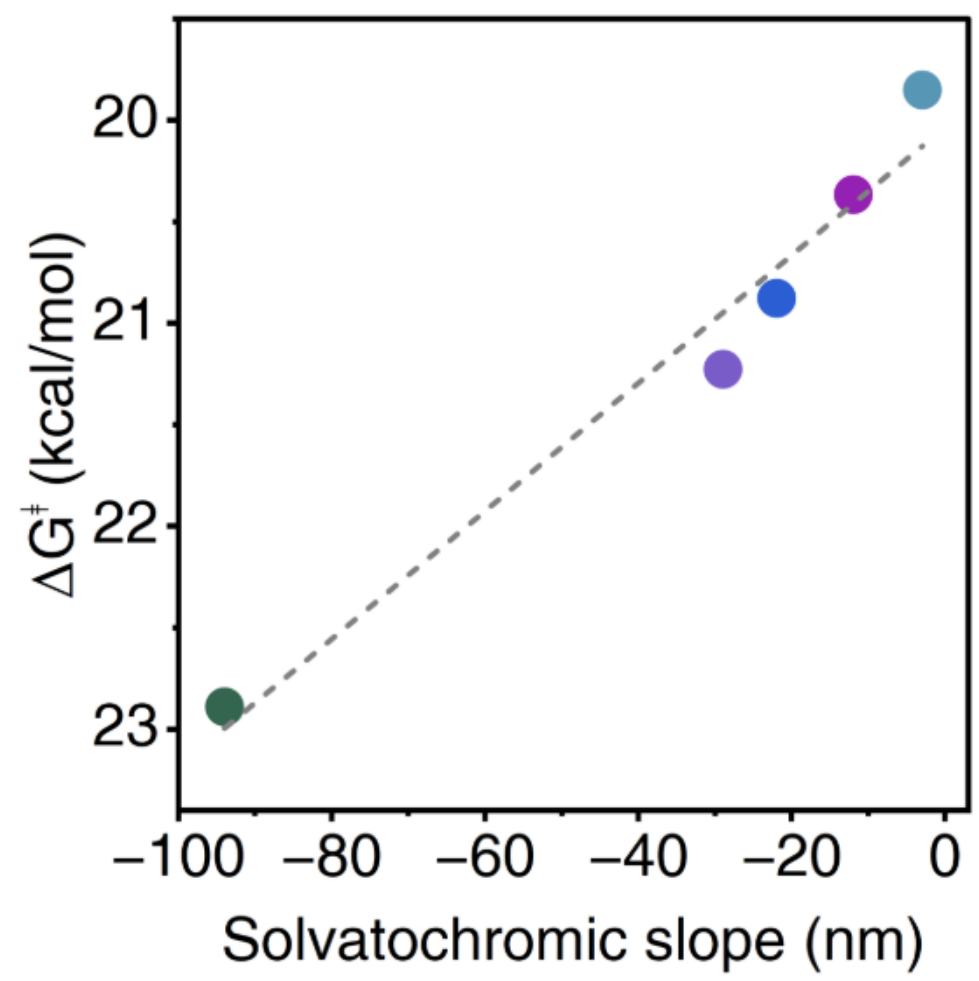

Figure S17. Linear correlation between activation free energy in LCE phase and solvatochromic slope from small molecule studies.

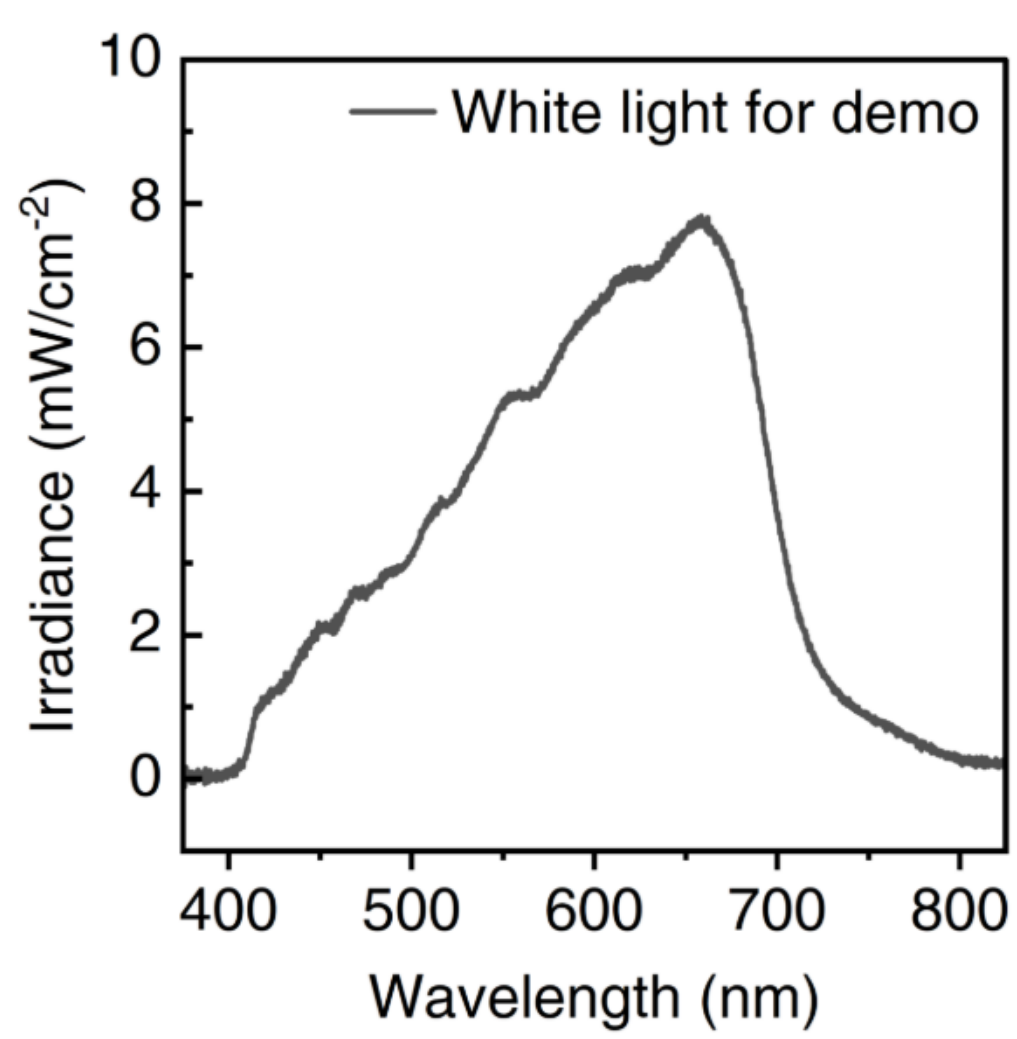

Figure S18. Spectral profile of the white light used for optical demonstrations for DASA-LCEs.

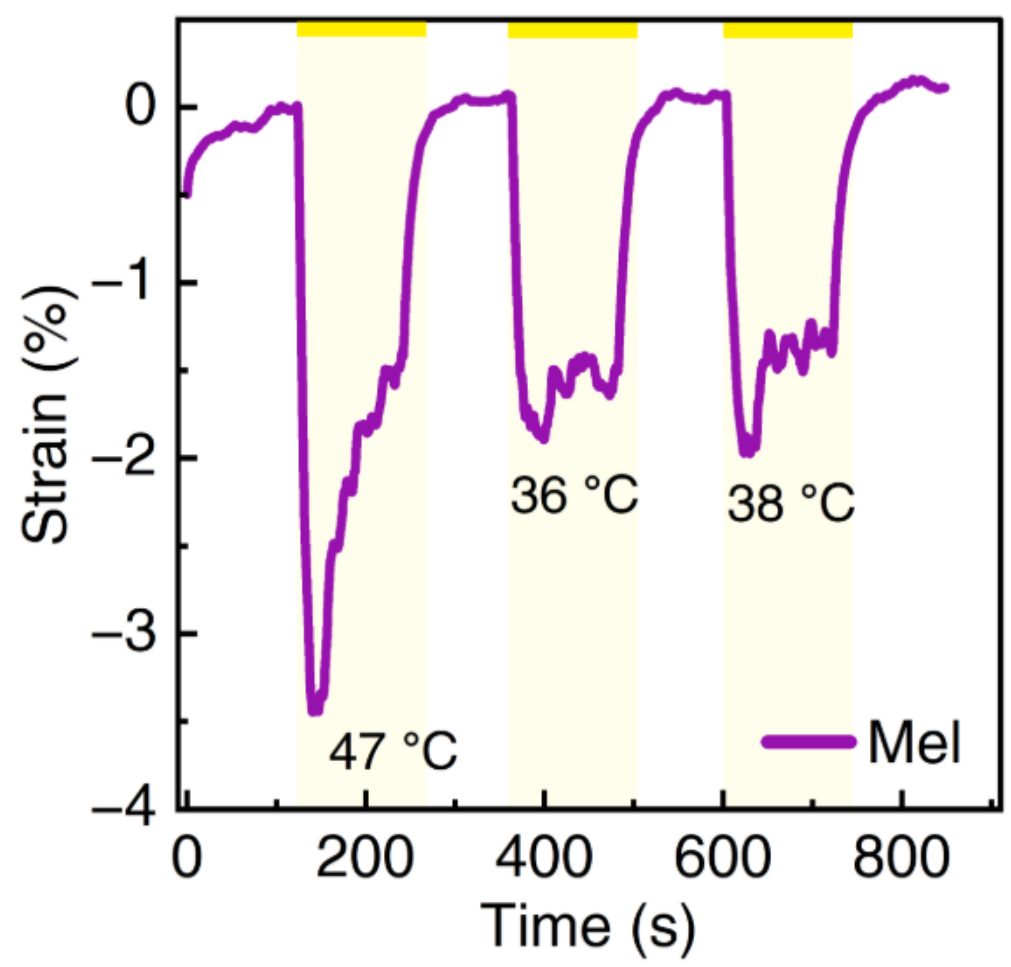

Figure S19. Strain trace of Mel-LCE under cyclic irradiation with IR camera temperature readouts at the maximum strain

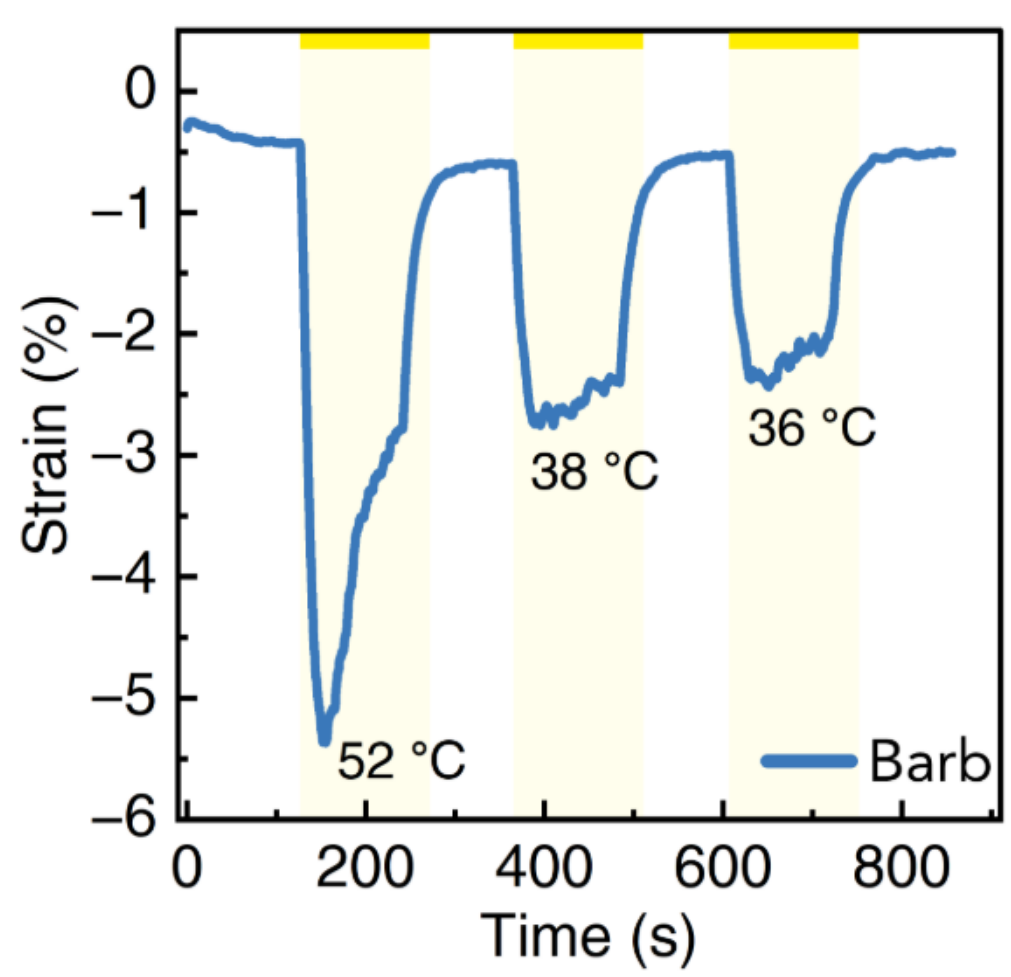

Figure S20. Strain trace of Mel-LCE under cyclic irradiation with IR camera temperature readouts at the maximum strain

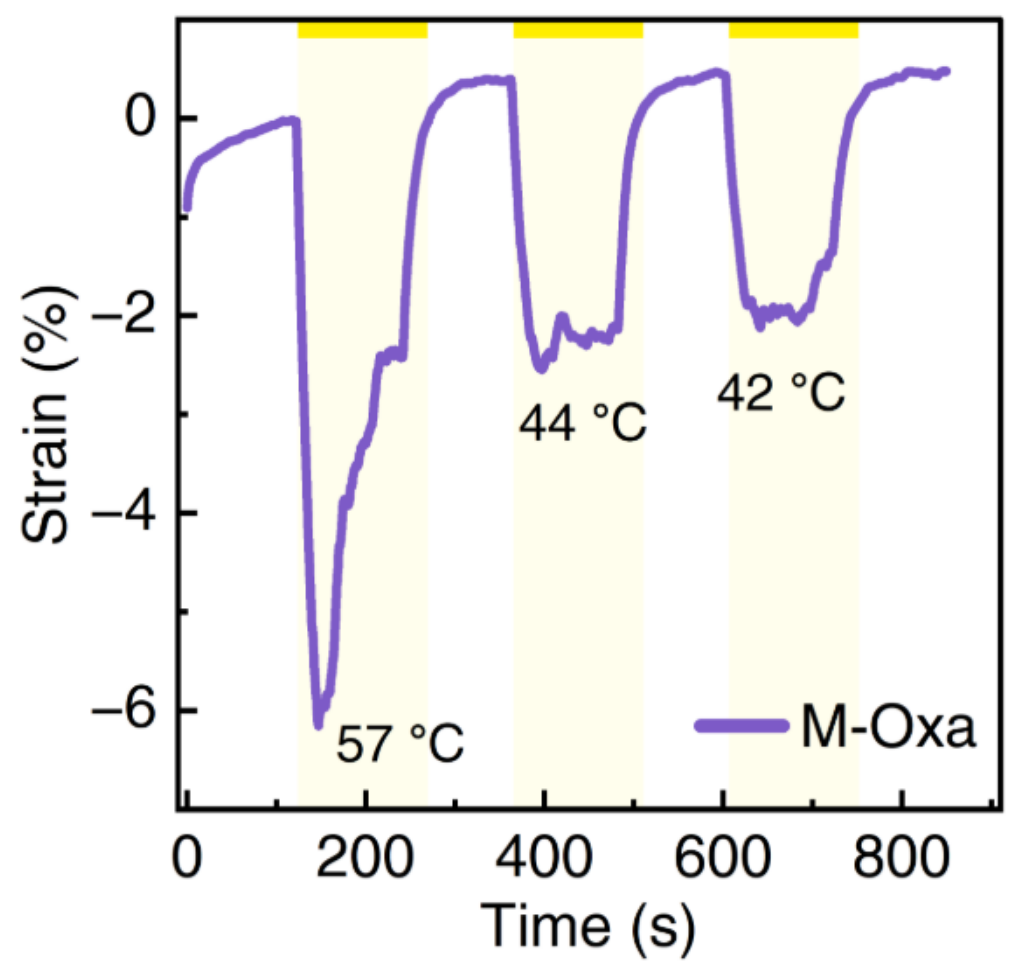

Figure S21. Strain trace of Mel-LCE under cyclic irradiation with IR camera temperature readouts at the maximum strain

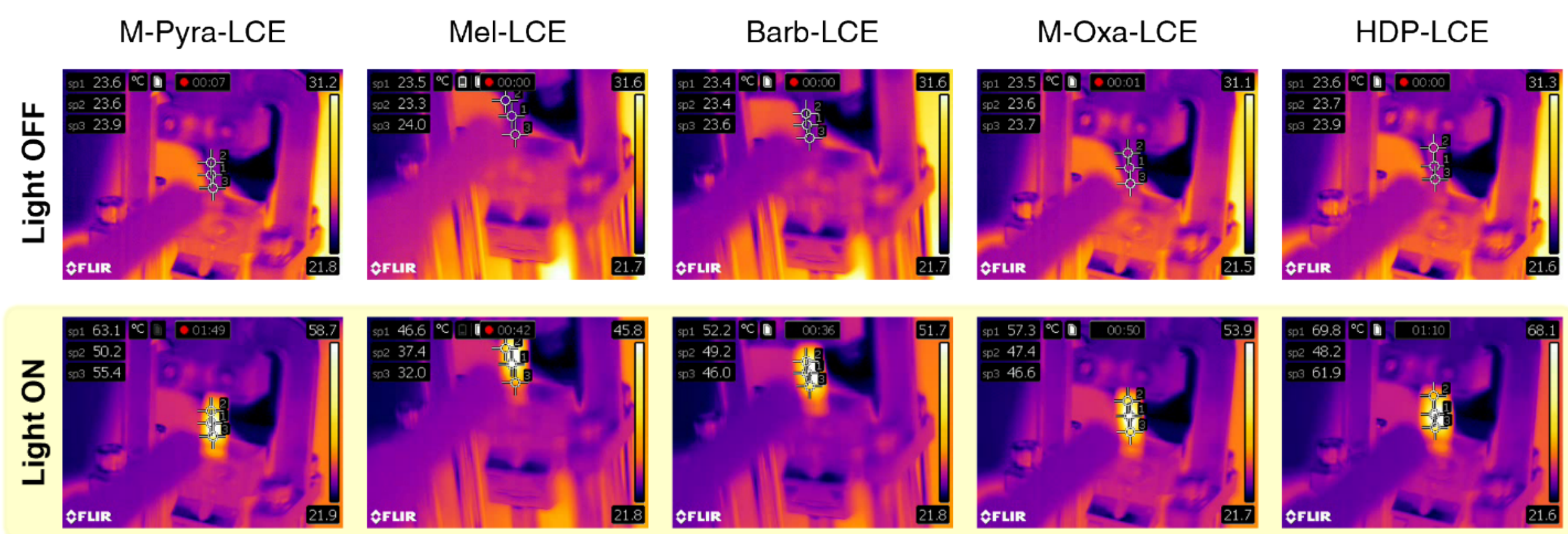

Figure S22. Strain trace of Mel-LCE under cyclic irradiation with IR camera temperature readouts at the maximum strain

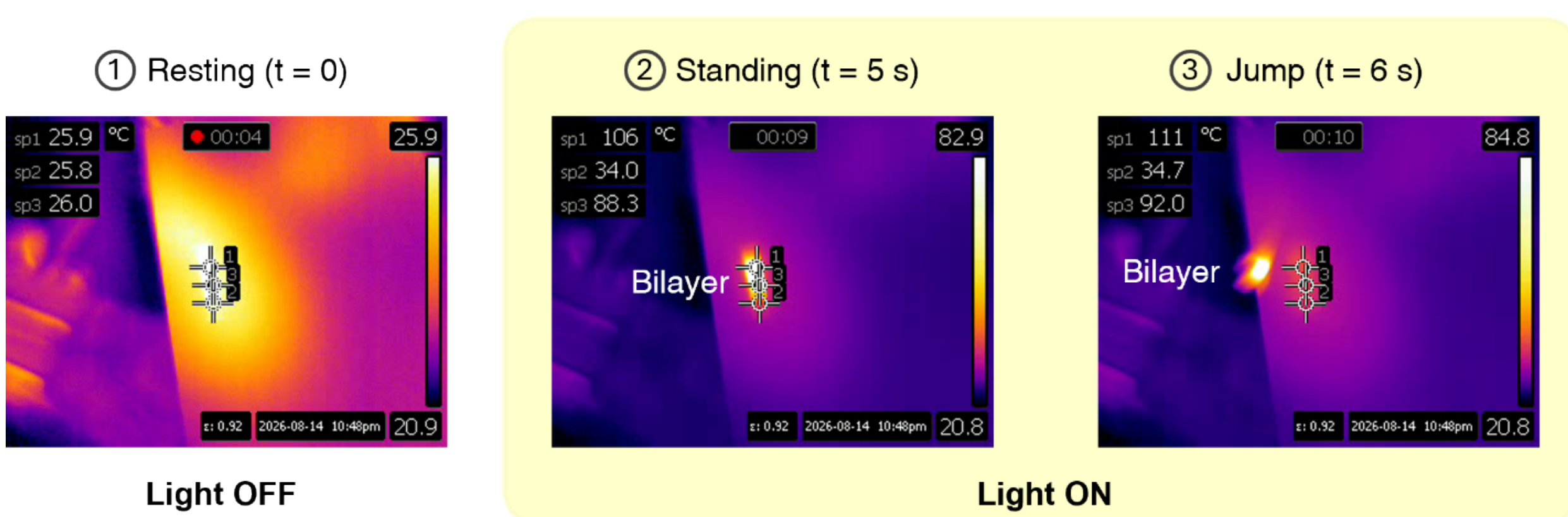

Figure S23. Strain trace of Mel-LCE under cyclic irradiation with IR camera temperature readouts at the maximum strain

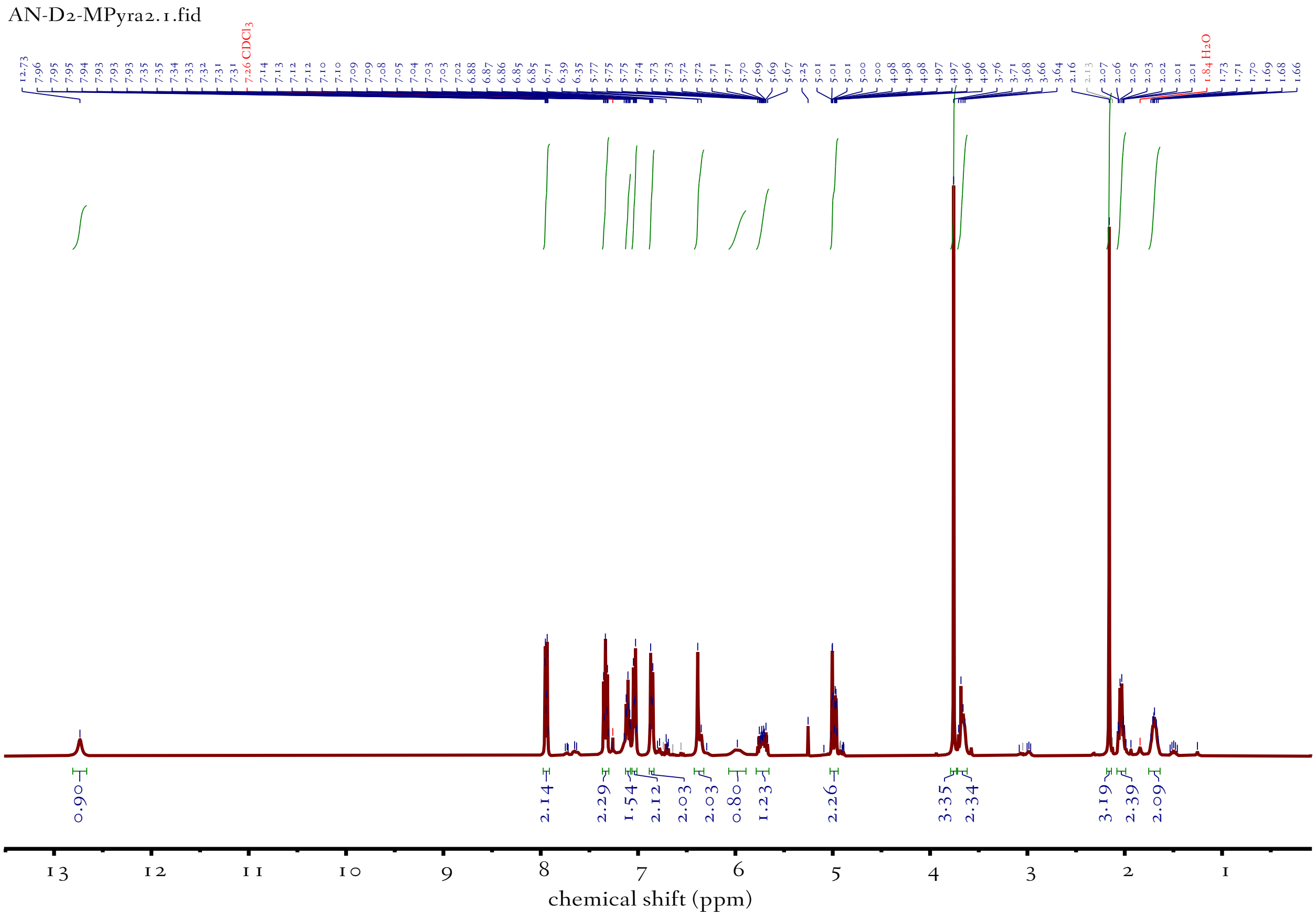

Figure S24. 1H NMR trace of the Methyl-pyrazolone derivative DASA small molecule used in the model systems in CD_2Cl_2.

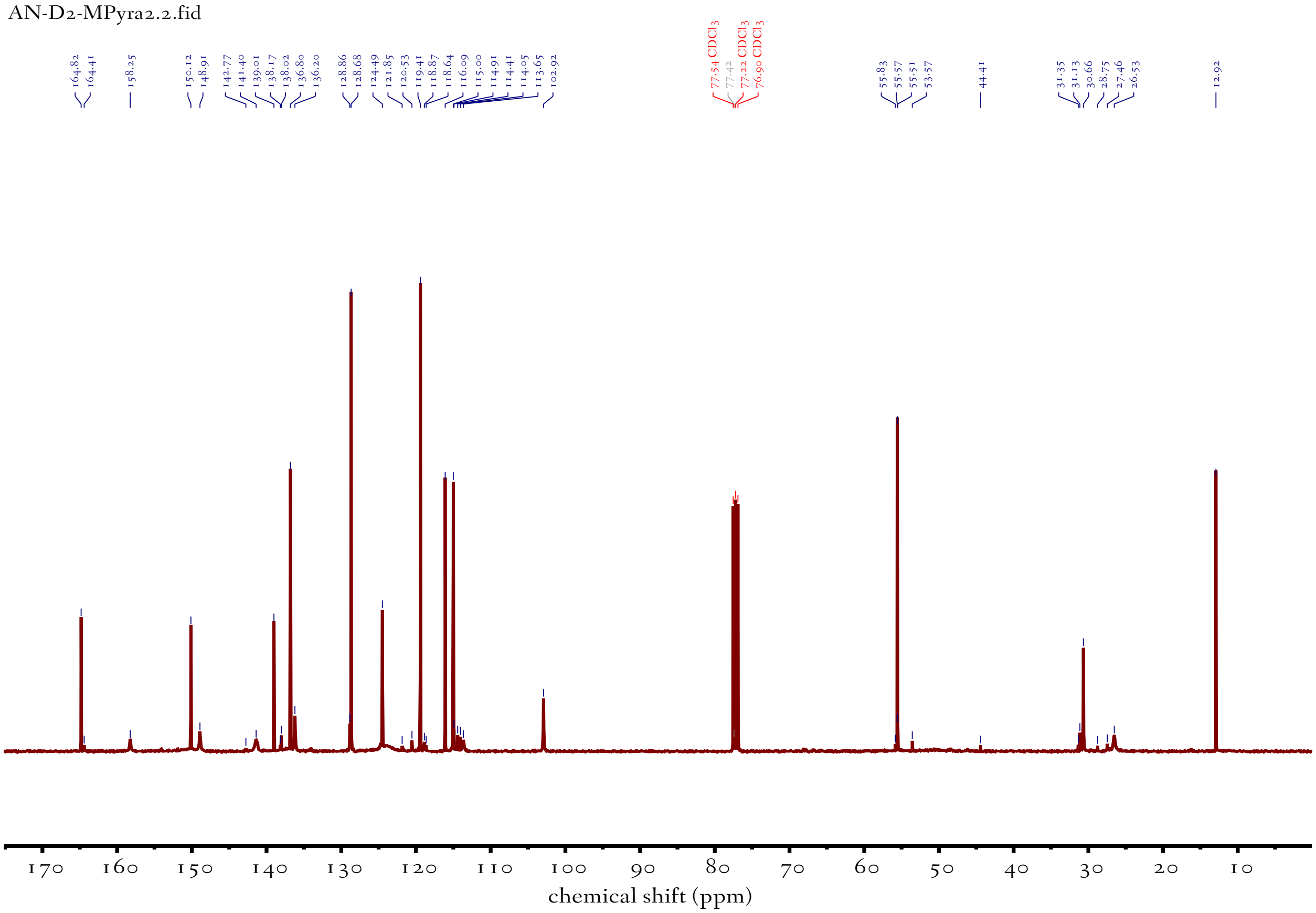

Figure S25. ^{13}C NMR trace of the Methyl-pyrazolone derivative DASA small molecule used in the model systems in CD_2Cl_2.

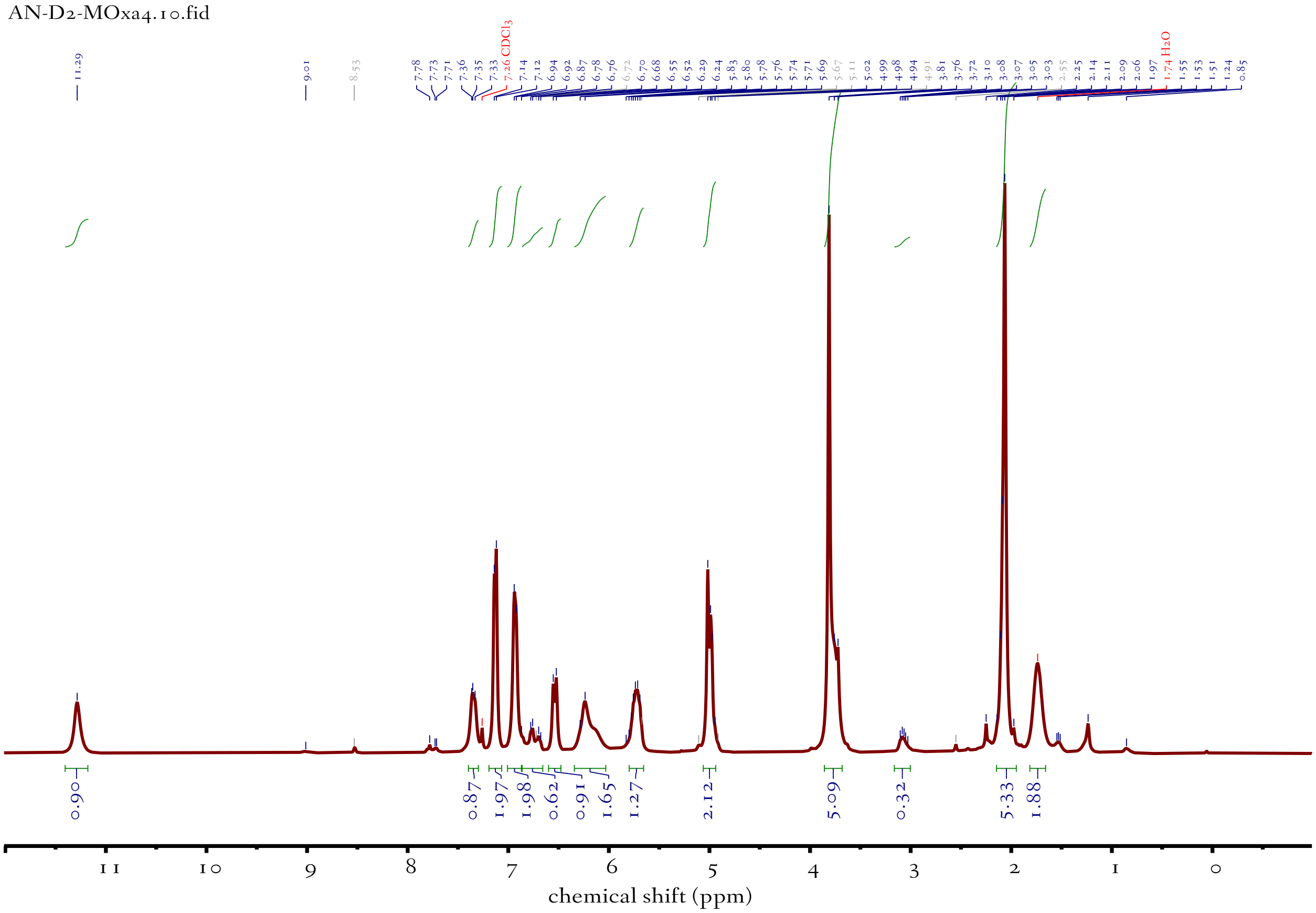

Figure S26. ^{1}H NMR trace of the Methyl-oxazolone derivative DASA small molecule used in the model systems in CD_2Cl_2.

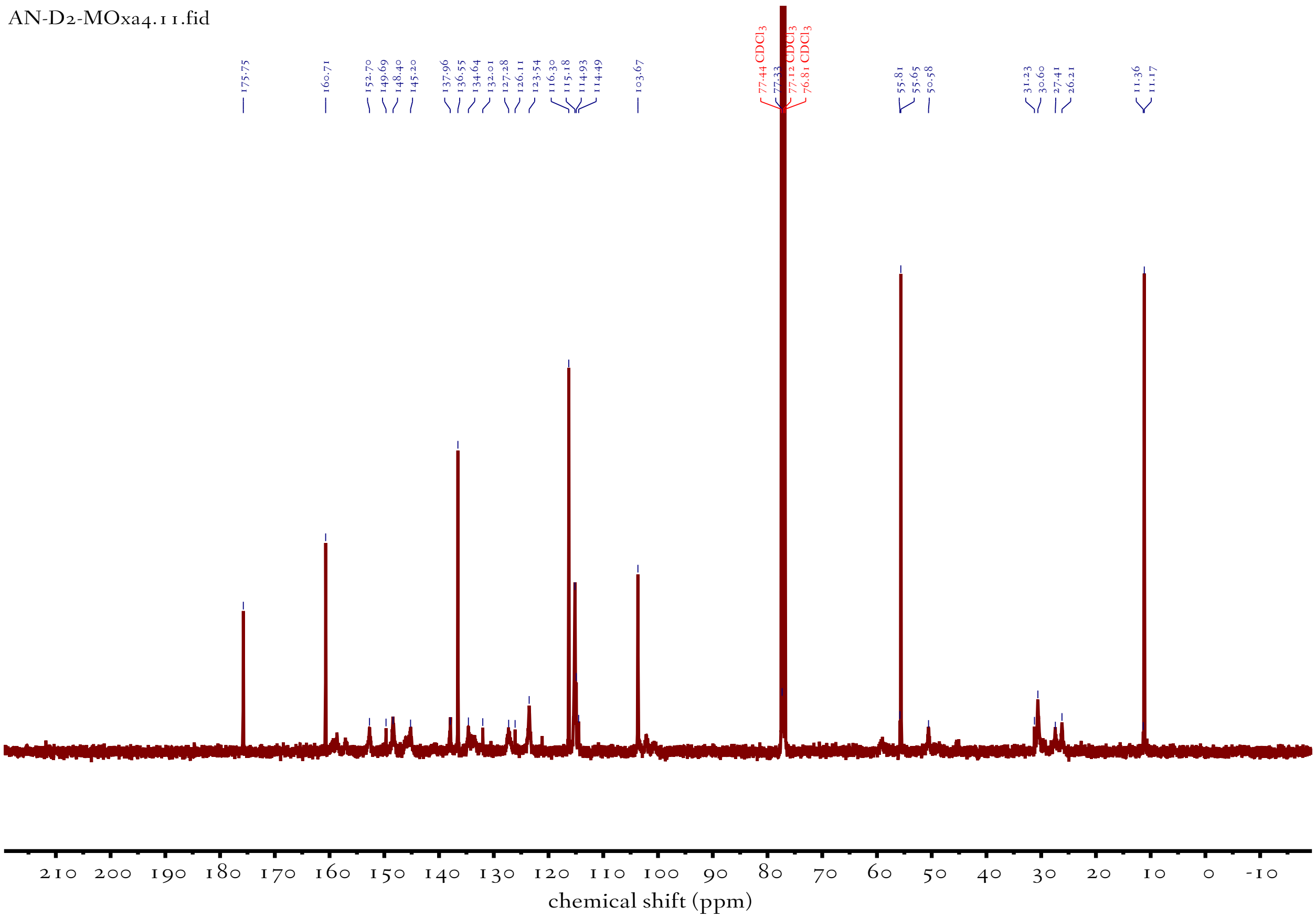

Figure S27. ^{13}C NMR trace of the Methyl-oxazolone derivative DASA small molecule used in the model systems in CD_2Cl_2.

References:

1 M. T. Sims, L. C. Abbott, R. M. Richardson, J. W. Goodby and J. N. Moore, Considerations in the determination of orientational order parameters from X-ray scattering experiments, *Liquid Crystals*, 2019, **46**, 11–24.

2 M. Clerc, C. Tekin, S. Ulrich, R. V. M. Freire, S. Salentinig, N. Bruns and L. F. Boesel, Donor–Acceptor Stenhouse Adduct-Polydimethylsiloxane-Conjugates for Enhanced Photoswitching in Bulk Polymers, *Macromol. Rapid Commun.*, 2022, **43**, 2200120.

3 S. Helmy, F. A. Leibfarth, S. Oh, J. E. Poelma, C. J. Hawker and J. Read de Alaniz, Photoswitching Using Visible Light: A New Class of Organic Photochromic Molecules, *J. Am. Chem. Soc.*, 2014, **136**, 8169–8172.

4 J. R. Hemmer, Z. A. Page, K. D. Clark, F. Stricker, N. D. Dolinski, C. J. Hawker and J. Read de Alaniz, Controlling Dark Equilibria and Enhancing Donor–Acceptor Stenhouse Adduct Photoswitching Properties through Carbon Acid Design, *J. Am. Chem. Soc.*, 2018, **140**, 10425–10429.

5 P. V. Khairnar, Y.-H. Su, A. Edukondalu and W. Lin, Enantioselective Synthesis of Spiropyrazolone-Fused Cyclopenta[*c*]chromen-4-ones Bearing Five Contiguous Stereocenters via (3+2) Cycloaddition, *J. Org. Chem.*, 2021, **86**, 12326–12335.
